# Genome-wide characterization of the C2H2 zinc-finger genes in *Cucumis sativus* and functional analyses of four *CsZFPs* in response to stresses

**DOI:** 10.1186/s12870-020-02575-1

**Published:** 2020-07-29

**Authors:** Junliang Yin, Lixin Wang, Jiao Zhao, Yiting Li, Rong Huang, Xinchen Jiang, Xiaokang Zhou, Xiongmeng Zhu, Yang He, Yiqin He, Yiqing Liu, Yongxing Zhu

**Affiliations:** 1 Hubei Key Laboratory of Waterlogging Disaster and Agricultural Use of Wetland/College of Agriculture, Yangtze University, Jingzhou, 434000 Hubei China; 2 College of Horticulture, Hebei Agricultural University, Baoding, 071001 Hebei China; 3 College of Horticulture and Gardening, Yangtze University, Jingzhou, 434000 Hubei China

**Keywords:** C2H2 zinc-finger proteins, Cucumber, Phylogeny, Stresses, Functional analysis

## Abstract

**Backgrounds:**

C2H2-type zinc finger protein (ZFPs) form a relatively large family of transcriptional regulators in plants, and play many roles in plant growth, development, and stress response. However, the comprehensive analysis of C2H2 ZFPs in cucumber (*CsZFPs*) and their regulation function in cucumber are still lacking.

**Results:**

In the current study, the whole genome identification and characterization of *CsZFPs*, including the gene structure, genome localization, phylogenetic relationship, and gene expression were performed. Functional analysis of 4 selected genes by transient transformation were also conducted. A total of 129 full-length *CsZFPs* were identified, which could be classified into four groups according to the phylogenetic analysis. The 129 *CsZFPs* unequally distributed on 7 chromosomes. Promoter *cis*-element analysis showed that the *CsZFPs* might involve in the regulation of phytohormone and/or abiotic stress response, and 93 *CsZFPs* were predicted to be targeted by one to 20 miRNAs. Moreover, the subcellular localization analysis indicated that 10 tested CsZFPs located in the nucleus and the transcriptome profiling analysis of *CsZFPs* demonstrated that these genes are involved in root and floral development, pollination and fruit spine. Furthermore, the transient overexpression of *Csa1G085390* and *Csa7G071440* into *Nicotiana benthamiana* plants revealed that they could decrease and induce leave necrosis in response to pathogen attack, respectively, and they could enhance salt and drought stresses through the initial induction of H_2_O_2_. In addition, *Csa4G642460* and *Csa6G303740* could induce cell death after 5 days transformation.

**Conclusions:**

The identification and function analysis of *CsZFPs* demonstrated that some key individual *CsZFPs* might play essential roles in response to biotic and abiotic stresses. These results could lay the foundation for understanding the role of *CsZFPs* in cucumber development for future genetic engineering studies.

## Background

Plants frequently suffer from various biotic and abiotic stresses which adversely affect plant growth and development [1, 2], while transcription factor (TFs) are important regulators that involved in various biological and environmental stress processes [3]. Among them, zinc finger proteins (ZFPs) account for a relatively large family of eukaryotic transcription factors [4]. In *Arabidopsis* and rice, nearly 15 and 13% transcription factors are ZFPs (176 and 189, respectively) [5, 6]. According to the number and order of the cysteine (Cys, C) and histidine (His, H) residues, the ZFPs could be classified into several subgroups, such as C2H2, C2HC, C2HC5, C3HC4, CCCH, C4, C4HC3, C6, and C8 [7, 8]. Among them, C2H2-type zinc finger proteins (C2H2-ZFPs) are classical ones which have been widely studied. The C2H2-type of zinc finger proteins, also referred to as the TFIIIA type zinc finger that contains two Cys and two His residues in the zinc finger domain, are described as CX2-4CX3FX5LX2HX3-5H, which form the conservative and best-characterized DNA-binding motif [9, 10]. In plants, C2H2 zinc finger proteins have the similar structures which differ from those in other eukaryotic organisms. The highly conserved QALGGH motif in the zinc finger helices could be detected in C2H2-ZFPs. According to the number and patterns of zinc fingers, the C2H2-ZFPs could be categorized into three groups, including triple-C2H2 (tC2H2) zinc fingers, multiple-adjacent-C2H2 (maC2H2) zinc fingers, and separated-paired-C2H2 (spC2H2) zinc fingers [11], and furthermore, each group could be divided into various subgroups [5]. In addition, C2H2-ZFPs display a wide range of structure and functions, from DNA or RNA binding to the involvement in protein-protein interactions [5], which fulfill their function as key transcriptional regulators to play important roles in adverse stresses, such as drought, low-temperature, salt, and oxidative stresses [10].

Genome identification and functional analysis of C2H2-ZFPs have been studied in several plants and whilst some stress related C2H2-ZFPs have been characterized, including 176 members in the *Arabidopsis* genome [5], 189 in rice (*Oryza sativa* L.) [6], 122 in wheat (*Triticum aestivum* L.) [7], 301 in *Brassica rapa* L. [12], 109 in *Populus trichocarpa* [13], 189 in foxtail millet (*Setaria italica* L.) [9], 321 in soybean (*Glycine max* L.) [14], and 211 in maize (*Zea mays* L.) [15], suggesting the C2H2-ZFPs are extensively involved in plant growth, development, and defense responses [16–19]. For example, in *Arabidopsis*, *AZF1* (*At5g67450*) and *AZF2* (*At3g19580*) could function as transcriptional repressors to repress the expression of osmotic stress- and ABA-repressive genes to negatively affect plant growth [20]. *ZAT18* (*At3g53600*) was transcriptionally induced by drought stress, and overexpression of *ZAT18* improved drought tolerance while mutation of *ZAT18* resulted in decreased plant tolerance to drought stress [19]. Moreover, high expression of *ZAT7* in roots tissues could positively improve salt stress tolerance by activating defense genes [21]. Meanwhile, the *Arabidopsis thaliana* SUPERMAN (SUP) protein, one of the best studied C2H2, has been shown to regulate carpel numbers and ovule development [22], and the SUP orthologous in cucumber (*CsSUP*, *Csa3G141870*) has been identified and studied which might function importantly in the female flower buds and ovules development [23]. In *Poncirus trifoliata* (L.) Raf., the expression of *PtrZPT2–1* was strongly induced by cold, drought, and salt stresses and overexpression of *PtrZPT2–1* in tobacco enhanced its cold, drought, and salt resistance through increasing the levels of osmotic regulatory solutes and decreasing the accumulation of H_2_O_2_ [24]. In soybean, the transcription of a C2H2-type zinc finger protein, *GmSCOF-1*, was induced by low temperature and abscisic acid (ABA) treatments, but not by dehydration or high salinity [25]. Furthermore, overexpression of *GmSCOF-1* increases expression levels of cold responsive genes to improve cold tolerance in transgenic plants. These results indicate that C2H2-ZFPs function as transcriptional activators or repressors and control the transcriptional levels of downstream genes under different stress signal transduction pathways. However, the comprehensive identification and stress related function analysis of C2H2-ZFPs in cucumber (*Cucumis sativus* L.) remain elusive.

Cucumber is an important vegetable crop cultivated worldwide [26]. The quality and yield of cucumber are easily affected by adverse environmental stresses, such as high drought, salinity, and nutrient deficiency, since its root largely spreads in shallow soil, [27]. In the past few years, the dramatically developed genome information and the completed genome sequencing of cucumber provide the opportunity for us to identify the interesting protein families which function importantly in various stresses at the genome-wide level [28]. Thus, to explore the function of cucumber *CsZFP* (*Cucumissativus*zinc finger protein) genes in response to biotic/abiotic stresses, we performed bioinformatics analysis to identify the main *CsZFP* members with further gene structure, genome localization, and phylogenetic relationship analysis. Then, the qRT-PCR analysis was conducted to abiotic stressed tissues, such as cold, salt, heat, and drought treated cucumber leaves and roots. Furthermore, the transient overexpression of selected *CsZFPs* into tobacco plants was performed and their subcellular localization, defense activation, and possible roles in response to drought, salt, and pathogen infection stresses was proceeded. This study provided clues for the functional characterization of *CsZFPs* and their potential application in cucumber molecular breeding related to stress tolerance.

## Results

### Genome-wide identification of ZFP genes in cucumber

Using multiple plants’ ZFP protein sequences as searching queries, a total of 144 non-redundant CsZFP sequences were identified from cucumber genome as potential aimed genes. The non-representative alternative splicing forms origin from same gene locus were treated as same gene and finally 129 *CsZFP* genes were retrieved from cucumber genome database (Table 1). SMART and Pfam analysis confirmed the presence of 235 C2H2 zinc finger domain in these 129 *CsZFP* genes. In contrast, the number of *CsZFP* was less than that was identified in *Arabidopsis* (176) [5], rice (189) [6], *Brassica rapa* L. (301) [12], foxtail mille (189) [9], soybean [14], and maize (211) [15], but more than that in wheat (122) [7], *Populus trichocarpa* [13]. Hence, the numbers of the ZFP gene family much differ among different species.

**Table 1:** Identification of ZPF genes in cucumber (CsZFPs)

Gene ID	aLength	bMW	cpI	dGRAVY	fLoc
Csa1G524710	195	21.67	5.58	− 0.704	Nucleus
Csa2G190780	180	20.50	8.87	− 0.882	Nucleus
Csa3G011750	194	21.70	5.8	− 0.693	Nucleus
Csa3G141870	187	20.97	9.23	−0.631	Chloroplast
Csa3G426920	144	15.98	6.27	−0.776	Chloroplast
Csa3G733980	271	30.06	5.34	−0.872	Nucleus
Csa3G902400	259	28.38	5.51	−0.754	Nucleus
Csa5G477610	102	12.07	9.82	−0.748	Chloroplast
Csa5G577350	213	22.73	6.36	−0.566	Nucleus
Csa5G603380	257	28.95	5.63	−0.874	Nucleus
Csa2G382580	224	24.63	9.37	−0.792	Nucleus
Csa2G404810	165	18.70	9.44	−0.562	Nucleus
Csa2G190770	179	20.15	5.95	−0.897	Nucleus
Csa3G141860	181	20.47	6.34	−0.774	Nucleus
Csa3G196420	269	29.58	5.02	−0.374	Chloroplast
Csa3G199020	205	23.20	5.99	−0.73	Nucleus
Csa3G509430	166	18.31	8.79	−0.655	Nucleus
Csa3G624040	151	17.21	8.78	−0.709	Nucleus
Csa3G799120	247	26.55	8.72	−0.68	Nucleus
Csa3G819820	219	23.93	9.16	−0.686	Chloroplast
Csa4G006510	252	26.99	7.83	−0.555	Nucleus
Csa4G652110	209	22.86	6.71	−0.743	Nucleus
Csa5G255670	237	25.90	6.71	−0.486	Nucleus
Csa5G372190	266	28.71	8.6	−0.786	Nucleus
Csa5G548160	249	26.87	6.59	−0.53	Nucleus
Csa5G583280	311	33.52	6.77	−0.714	Nucleus
Csa5G609820	229	24.07	8.35	−0.354	Chloroplast
Csa6G312040	138	14.76	9.96	−0.437	Chloroplast
Csa6G492290	186	20.67	9.24	−0.704	Nucleus
Csa7G071620	209	23.25	9.41	−0.643	Nucleus
Csa7G406990	173	18.65	8.75	−0.68	Cell wall
Csa3G001720	242	26.22	5.62	−0.912	Nucleus
Csa6G118360	164	18.08	8.34	−0.504	Nucleus
Csa1G002750	280	32.27	8.97	−0.496	Nucleus
Csa2G174080	177	18.92	8.67	−0.216	Chloroplast
Csa3G035880	418	47.40	9.23	−0.589	Chloroplast
Csa3G043950	245	27.14	9.03	−0.978	Nucleus
Csa3G043960	263	28.68	6.24	−0.751	Chloroplast
Csa3G134950	241	26.48	5.86	−0.539	Nucleus
Csa3G748810	318	34.61	4.7	−1.162	Nucleus
Csa3G872110	424	45.89	9.51	−0.446	Chloroplast
Csa4G641580	420	48.17	6.79	−0.667	Chloroplast
Csa5G289630	141	15.59	9.84	−0.95	Chloroplast
Csa5G375760	427	46.71	9.42	−0.488	Chloroplast
Csa5G625970	627	70.64	4.69	−0.409	Chloroplast
Csa6G128030	525	59.31	8.37	−0.493	Nucleus
Csa6G150540	296	31.79	4.9	−0.88	Nucleus
Csa6G150550	296	31.71	4.69	−1.034	Nucleus
Csa6G516660	354	40.14	8.12	−0.582	Nucleus
Csa7G432050	229	25.40	6.31	−0.574	Nucleus
Csa2G033890	202	23.60	9.4	−1.25	Nucleus
Csa1G560650	156	17.87	8.27	−0.789	Nucleus
Csa3G603040	508	59.55	5.13	−0.83	Chloroplast
Csa6G446540	119	13.66	9.68	−0.863	Chloroplast
Csa2G292740	305	34.49	5.54	−1.06	Endoplasmic reticulum
Csa1G600970	590	65.81	5.77	−0.885	Chloroplast
Csa1G657510	105	11.31	9.84	−0.849	Nucleus
Csa1G008490	1608	181.16	5.47	−0.628	Nucleus
Csa1G560720	299	34.33	6.38	−1.129	Nucleus
Csa3G098560	435	46.83	9.43	−0.39	Chloroplast
Csa3G125000	676	75.04	8.32	−0.707	Nucleus
Csa3G172410	245	27.14	9.03	−0.978	Nucleus
Csa3G182760	812	91.22	8.67	−0.669	Nucleus
Csa3G406040	443	48.03	9.44	−0.385	Chloroplast
Csa3G592150	281	32.78	9.52	−0.555	Chloroplast
Csa3G732590	471	51.28	6.35	−0.744	Nucleus
Csa6G509590	206	21.98	4.97	−1.15	Nucleus
Csa1G481720	155	17.39	9.11	−0.575	Chloroplast
Csa2G326490	225	25.01	8.99	−0.843	Nucleus
Csa2G354820	230	24.82	8.97	−0.601	Nucleus
Csa3G743410	257	28.37	7.64	−0.648	Nucleus
Csa4G293330	162	17.90	9.22	−0.317	Nucleus
Csa4G642460	262	28.06	6.89	−0.674	Nucleus
Csa5G183770	131	15.00	9.8	−0.737	Nucleus
Csa6G148300	286	31.49	8.17	−0.276	Chloroplast
Csa6G303740	139	14.98	7.03	−0.361	Nucleus
Csa7G071440	253	27.60	9.01	−0.852	Nucleus
Csa5G622500	322	35.61	6.3	−0.817	Nucleus
Csa5G180920	181	19.75	9.42	−0.613	Nucleus
Csa4G166950	289	31.81	8.7	−0.541	Nucleus
Csa3G043940	200	22.39	9.31	−0.915	Nucleus
Csa7G024090	291	32.97	6.9	−0.831	Nucleus
Csa2G000290	316	34.88	9.24	−0.818	Chloroplast
Csa2G035540	420	47.76	9.11	−0.733	Nucleus
Csa3G731900	84	9.94	5.65	−0.658	Nucleus
Csa2G139840	411	46.23	5.96	−0.798	Nucleus
Csa3G810580	353	40.99	6.16	−1.067	Nucleus
Csa4G571220	335	37.29	6.27	−0.834	Nucleus
Csa5G092930	399	44.75	8.96	−0.693	Nucleus
Csa6G526390	372	39.98	8.24	−0.337	Nucleus
Csa3G019980	252	28.21	8.83	−0.843	Nucleus
Csa5G162030	263	29.84	9.39	−0.892	Nucleus
Csa3G117400	369	41.83	5.53	−1.089	Nucleus
Csa3G117410	564	62.81	6.08	−0.871	Nucleus
Csa5G606610	447	50.58	9.28	−1.058	Nucleus
Csa6G502050	507	53.64	8.69	−0.511	Nucleus
Csa7G039240	425	47.38	9.07	−0.67	Nucleus
Csa7G387730	483	52.68	9.27	−0.698	Nucleus
Csa7G395240	221	25.28	9.51	−1.029	Nucleus
Csa7G450800	551	59.45	8.89	−0.683	Nucleus
Csa1G029620	454	50.48	8.83	−0.852	Nucleus
Csa1G085390	375	41.55	9.4	−0.575	Chloroplast
Csa1G132120	375	42.34	9.1	−0.698	Nucleus
Csa1G160590	308	34.85	8.7	−0.743	Nucleus
Csa1G569480	448	49.03	8.88	−0.53	Nucleus
Csa2G361490	458	47.18	8.98	−0.221	Chloroplast
Csa2G409480	422	46.80	9.08	−0.614	Nucleus
Csa3G738980	499	54.14	9.39	−0.612	Nucleus
Csa3G848250	527	58.54	9	−0.809	Nucleus
Csa4G038810	405	43.25	9.15	−0.381	Nucleus
Csa4G095040	355	40.37	7.86	−0.818	Nucleus
Csa4G646130	408	45.43	9.12	−0.722	Nucleus
Csa5G270900	618	65.87	9.16	−0.754	Nucleus
Csa6G445020	448	49.57	9.35	−0.624	Nucleus
Csa5G365160	286	32.29	7.93	−0.821	Nucleus
Csa1G043020	424	48.84	9.07	−1.026	Nucleus
Csa2G406660	249	28.30	8.77	−0.68	Nucleus
Csa4G290830	353	39.92	7.41	−0.866	Nucleus
Csa7G290450	512	56.58	5.86	−0.605	Nucleus
Csa7G428260	293	32.84	8.63	−0.676	Chloroplast
Csa1G012120	481	54.06	8.53	−0.895	Nucleus
Csa1G039050	505	56.60	8.51	−0.89	Nucleus
Csa6G487810	1463	164.89	6.97	−0.507	Nucleus
Csa6G499870	376	41.69	5.7	−0.401	Nucleus
Csa1G012110	445	48.11	9.39	−0.439	Nucleus
Csa3G881730	395	44.86	5.47	−0.742	Nucleus
Csa7G419600	824	91.41	7.92	−0.784	Nucleus
Csa5G587130	370	42.49	6.18	−1.108	Chloroplast
Csa5G623900	402	45.78	8.5	−0.642	Nucleus

^1^ Length: length of amino acid; ^2^*MW* molecular masses, ^3^*pI* isoelectric point, ^4^*GRAVY* Grand average of hydrophobicity, ^5^*Loc* best possible cell localization predicted by the WoLF PSORT tool

Furthermore, the detailed physiological and biochemical information of these *CsZFPs*, including gene ID, length of amino acid (aa), molecular masses (MW), isoelectric point (pI), and predicted subcellular localization of each proteins were shown in Table 1. Independently, the amino acid length of each CsZFP differs from 84 aa (Csa3G731900) to 1608 aa (Csa1G008490) and the average length is 338 aa. The MWs of CsZFPs ranged from 9.93 (Csa3G731900) to 181.16 (Csa1G008490) kDa with the average 37.59. The pI values, an important physicochemical property of proteins, ranged from 9.96 (Csa6G312040) to 4.69 (Csa5G625970, Csa6G150550), respectively. Then, in silico prediction suggested the most majority of CsZFPs (99) with a subcellular localization at nucleus, 28 CsZFPs located at chloroplast, one located at cell wall (Csa7G406990), and one located at endoplasmic reticulum (Csa2G292740), which was consistent with the prediction of ZFPs in maize that most were located at the nucleus except several were at the chloroplast, cytosol, or mitochondria [15].

### Chromosomal localization, gene duplication, and phylogenetic analysis of CsZFP genes

**Fig. 1:**
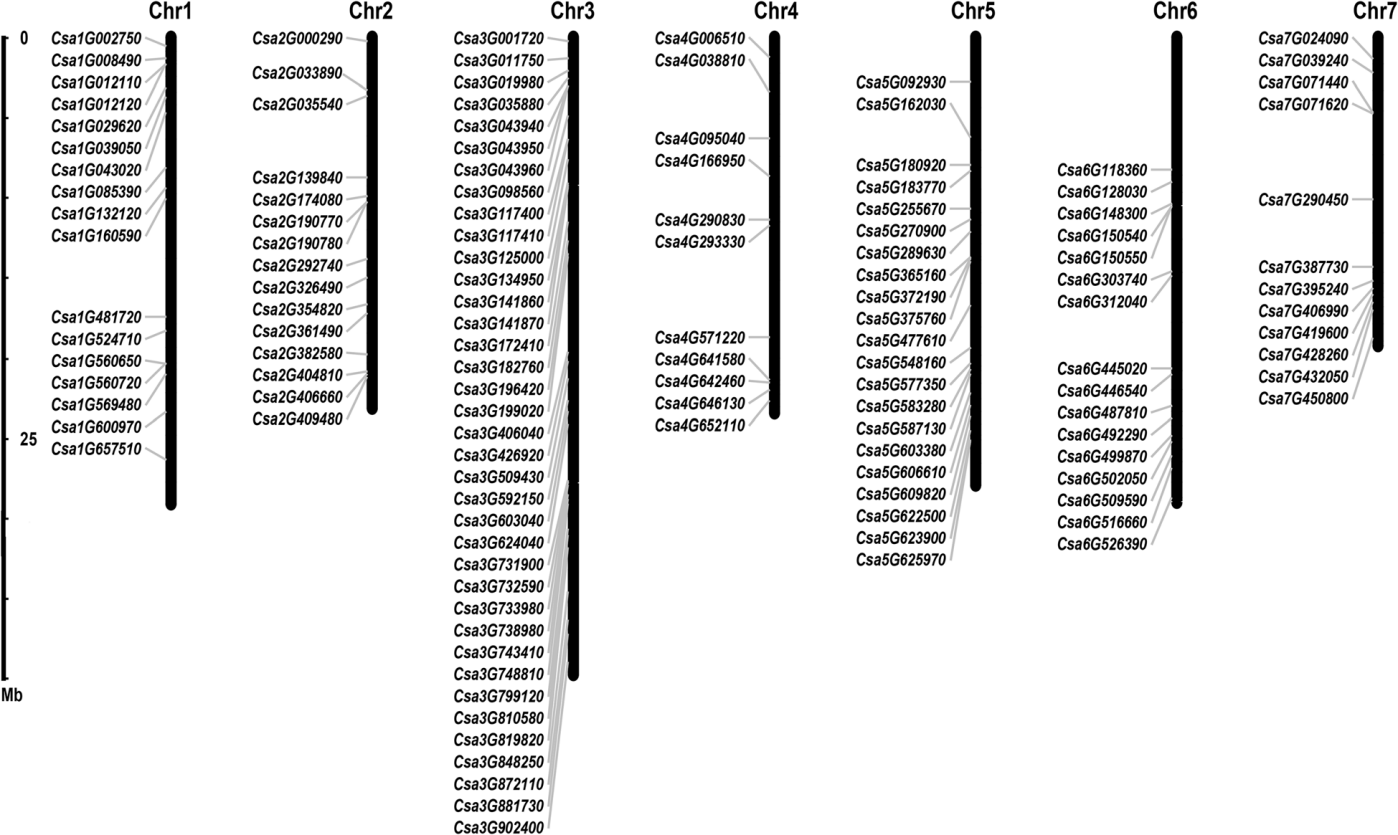
Chromosomal distribution of 129 C2H2 zinc-finger proteins (*CsZFPs*) on cucumber 7 chromosomes. Chr1–7 represent the chromosome 1 to 7. The rule on the left indicates the physical map distance among genes (Mb). The figure was drawn by Junliang Yin

To get the global view about the distribution of C2H2-ZFP genes in cucumber, the chromosomal location of 129 *CsZFPs* on 7 chromosomes was shown in Fig. 1, that 17, 15, 37, 11, 21, 16, and 12 *CsZFP*s were presented on Chr1 (Chromosome 1), Chr2, Chr3, Chr4, Chr5, Chr6, and Chr7, respectively. Among them, Chr3 contained a maximum number of *CsZFP* genes (37) and Chr4 contained the minimal number of *CsZFP* genes (11), which demonstrated *CsZFP*s were widely but unevenly distributed across 7 cucumber chromosomes. In addition, gene duplication analysis revealed that no segmental and tandem duplication took place during the evolutionary process of cucumber genome. Comparing with *Arabidopsis* (176) and rice (189), the smaller number of *CsZFPs* might be attributed to the absence of segmental duplication events in cucumber genome [5, 6].

To evaluate the phylogenetic relationship of CsZFPs, the amino acid sequences were used to construct an un-rooted phylogenetic tree through multiple sequence alignment. According to the plant-specific amino acid residues and distances between conserved motif sequence as described in soybean and *Populus trichocarpa* [13, 14], the C2H2-ZF domains in CsZFPs can be classified into five main types, as shown in Additional file 1: Table S1: (i) Domains contained plant-specific conserved amino acid sequence “QALGGH” and a conserved spacing “X2-C-X2-C-X7-QALGGH-X3-H” were designated as Q-type, where X is any amino acid and the number indicates the consensus spacing between the conserved amino acid residues. (ii) Domains with certain modifications of the Q-type ZF, including 1 to 5 degraded amino acids in the motif “QALGGH” and certain modifications in the spacing between the two cysteines (Cys, C) and two histidines (His, H), were classified as the M-type; Based on conserved motif sequence and conserved spacing of amino acids, M-type ZF were further divided into M1 to M13 subtypes. (iii) The C2H2-ZFPs domains with more than 12 (Z1-type) and less than 12 (Z2-type) amino acid between the second Cys and the first His were classified as Z-type. (iv) The C2H2-ZFPs domains with 12 amino acid between the second Cys and the first His were classified as the C-type. (v) The D-type did not contain the second His in the C2H2-ZF region.

**Fig. 2:**
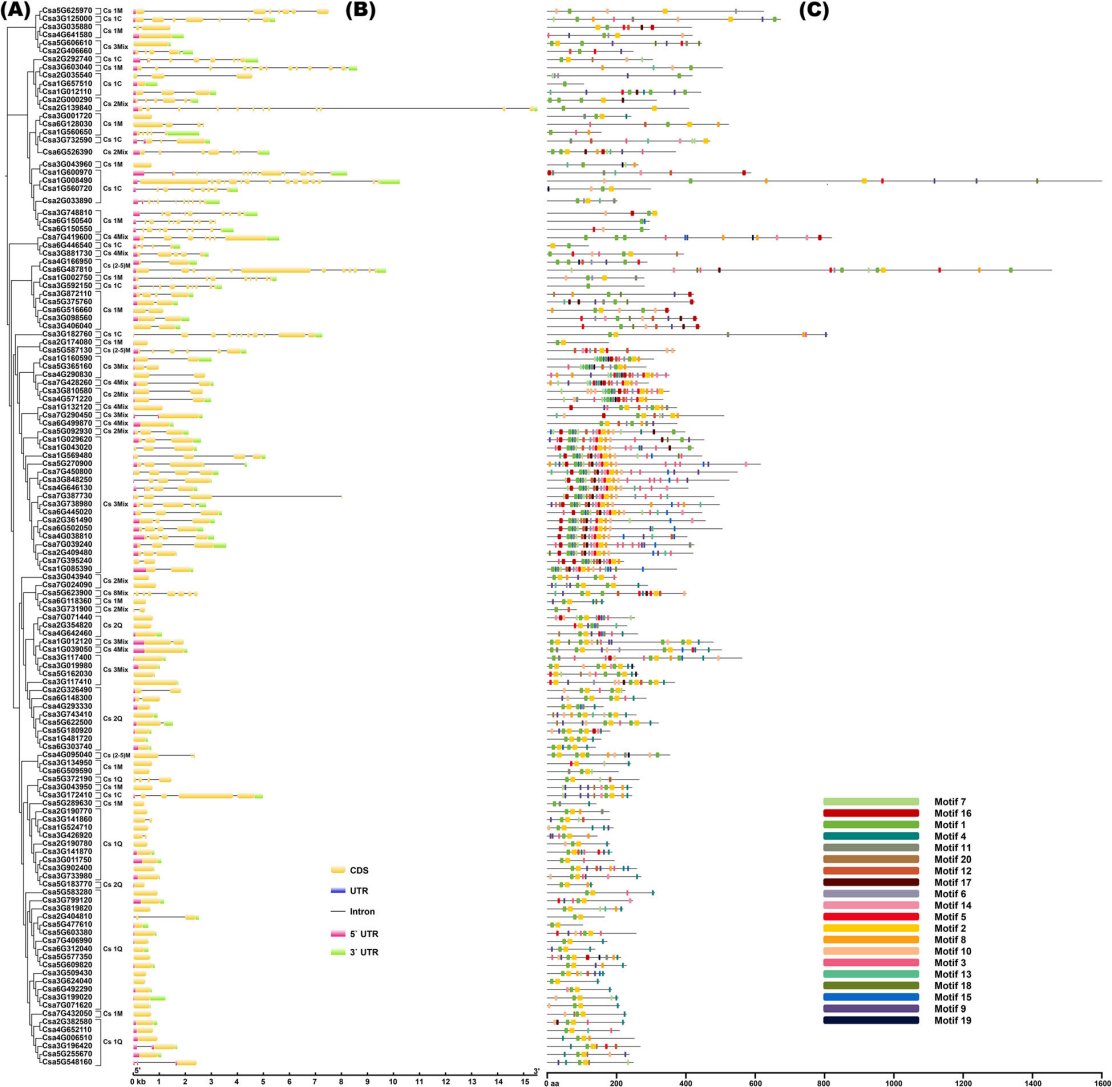
The phylogenetic tree, exon and intron distribution and motif analysis of CsZFPs. **a** The phylogenetic analysis of CsZFP protein sequences. The MEGA7 was used to do the phylogenetic analysis with the neighbor-joining method. **b** Schematic diagrams of the gene structures of *CsZFPs*. **c** The motif compositions and motif logos of CsZFPs in corresponding to the phylogenetic tree. The figure was drawn by Junliang Yin

Furthermore, based on the types and numbers of C2H2-ZF domains, CsZFPs were further classified into nine groups as shown in Fig. 2a and Additional file 1: Table S2: (1) Cs1Q (the 1st group of Q-type CsZFP, containing 31 members) and Cs2Q (the 2nd group, containing 12 members), which contains one and two conserved motif “QALGGH” in their protein sequences, respectively. (2) Cs1M group contain two to five M-type conserved motif sequence, and 24 CsZFPs were classified into Cs1M. (3) Cs(2–5) M group contain two to five M-type conserved motif sequence, and 4 members were classified into this group. (4) Cs1C (the 1st group of C-type CsZFP) contains one C-type conserved motif sequence. And 14 CsZFPs were classified into Cs1C. (5) Cs2Mix (mixture), Cs3Mix, Cs4Mix, and Cs8Mix groups contain mixture types of conserved motif sequence. And 9, 29, 5, and 1 CsZFPs could be clustered into Cs2Mix, Cs3Mix, Cs4Mix, and Cs8Mix, respectively. In summary, Cs1Q contained the largest number of the C2H2-ZF genes (31), followed by Cs3Mix (29) and Cs1M (24), and only one gene (Csa5G623900) belonged to Cs8Mix.

### Gene structure and motifs assay

The divergence of gene structure (e.g. the exon-intron and conserved motifs diversity) provides the potential insights of gene function of evolution [29]. As shown in Fig. 2b, the number of introns varied from 0 (*Csa7G432050*) to 14 (*Csa1G008490*). In addition, the most closely related members in same groups shared similar exon/intron structure in terms of intron number, exon length and/or location (Fig. 2b). For example, most *CsZFPs* in Cs1Q group have none introns, whereas only the *Csa2G404810* contained two introns and the same gene structure could be observed in Cs2Q group. While the largest number of introns could be found in Cs2Mix, Cs3Mix, Cs4Mix, and Cs8Mix groups with striking distinctions. These results showed that the gene structure changes of the CsZFPs might play important role in their function divergences during the evolution process.

The MEME program was used to predict putative conversed motifs in the CsZFP sequences of cucumber, and as showed in Fig. 2c, 20 distinct motifs were identified. Generally, motif 1, 4 and motif 5, 6, 14 formed the two typical C2H2 domains, and one or both of them were found to be existed in CsZFP proteins to form the conserved reigns of finger-like fold. Motif 1 contained a plant-specific conserved “QALGGH” in the zinc finger helices, which was previously classified as the Q-type zinc finger motif and was reported playing key roles in the recognition of specific DNA bases [5]. In addition, 43 out of 129 CsZFPs had the conserved “QALGGH” motif in the zinc finger helices. Motif 2, 8 and 10 formed the SprT-like zinc ribbon domain, which was function as Zn^+^ binding zone (Additional file 1: Table S1, 2, Additional file 2: Fig. S1). Finally, most closely related members in the CsZFP phylogenetic tree had similar motif compositions, suggesting functional similarities among the CsZFP proteins within the same group.

### Prediction of cis-acting regulatory elements

**Fig. 3:**
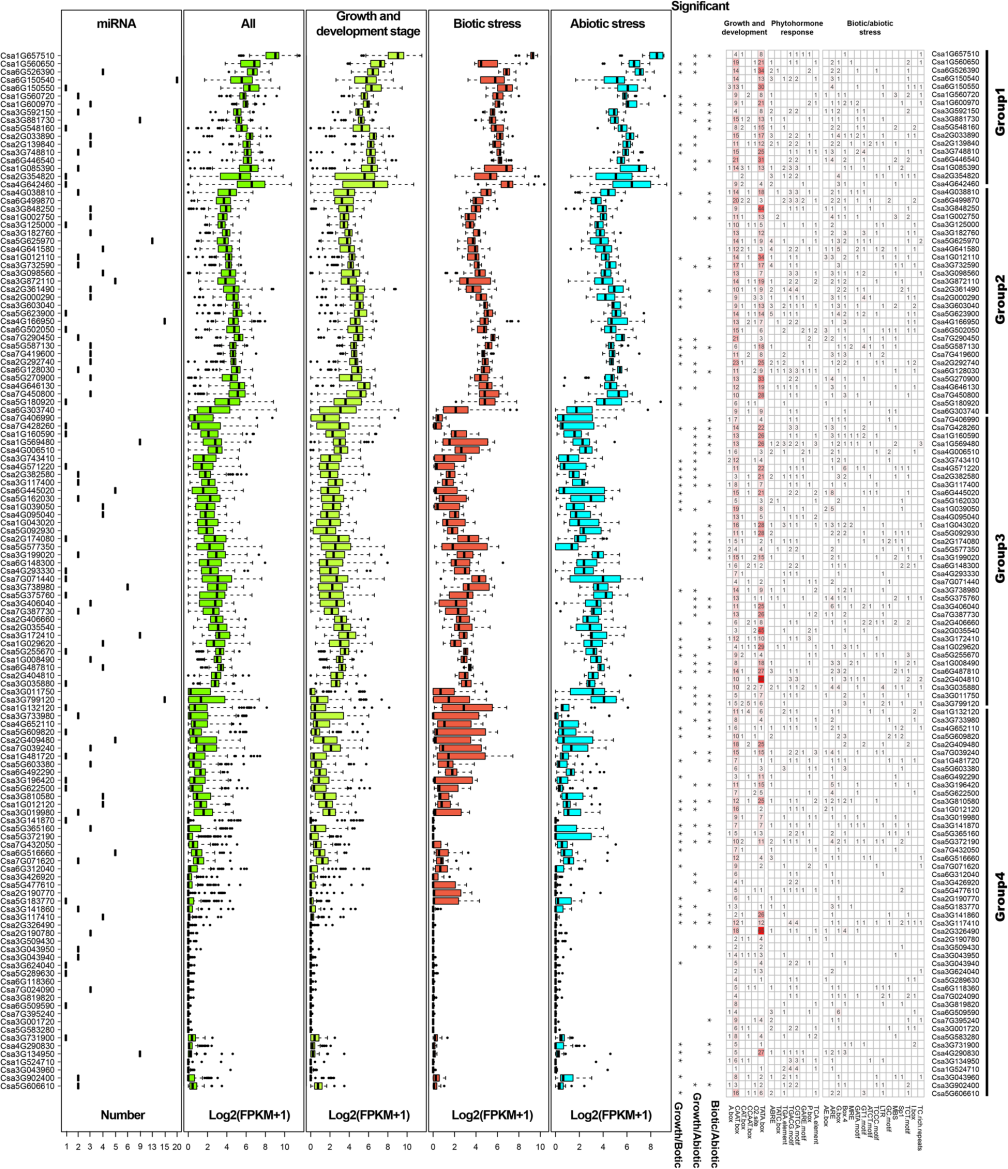
The number of miRNAs targeted to *CsZFPs*, box figure of *CsZFPs* expressing levels and the number of *cis*-regulatory elements of *CsZFPs* promoter region in response cucumber growth, development and biotic and abiotic stresses. The figure was drawn by Junliang Yin

To better understand the function of *CsZFPs*, especilally under biotic and abiotic stresses conditions, 1.5 kb non-coding sequences upstream of the CsZFPs translation start site (TSS), which belong to the promoter region, were adopted to predict the *cis*-regulatory elements within PlantCARE database (Fig. 3 and Additional file 1: Table S3, 4). Results showed that the promoter sequences of *CsZFPs* have various *cis*-regulatory elements, such as CAAT-box, CCAAT-box, and TATA-box, which existed extensively in all the promoter regions, suggesting the *CsZFPs* involved in the regulation of plant growth and development. Moreover, the *cis*-regulatory elements response to biotic and abiotic stresses and phytohormone were also detected in corresponding *CsZFPs*, including the ABRE (abscisic acid-responsive element), CGTCA-motif, TATC-box, TGACG-motif (elements responsive to MeJA), and TCA-element (salicylic acid-responsive element), which were response to phytohormone, and AE-box, G-box, MBS and LTR etc., which were related to biotic and abiotic conditions. In addition, according to the expression level of *CsZFPs* during growth and development process and in response to stresses, the *CsZFPs* could be classified into four groups. Moreover, these group 1 *CsZFPs* with higher expressing levels during growth and development stages, as well as biotic and abiotic stresses, commonly contained more *cis*-regulatory elements in their promoter regions. The opposite results were found in group 4 (Fig. 3), that lower expressed *CsZFPs* contained less *cis*-elements, demonstrating the number of *cis*-regulatory elements play important role in regulating the expression of *CsZFPs*.

### The miRNAs targeting CsZFPs transcripts

The miRNAs are highly conserved non-coding RNA which could regulate the translation of their target mRNAs [30]. Therefore, in order to get insights to the post-transcriptional adjustment of *CsZFPs*, the potential miRNAs targeting *CsZFPs* transcripts were predicted. As shown in Fig. 3 and Additional file 1: Table S5, many miRNAs could target to a total of 93 *CsZFPs* transcripts. The number of miRNAs target to corresponding *CsZFPs* transcripts ranges from 1 to 20. For example, the transcript of *Csa2G382580*, nine miRNAs target to it, including csa-miR-n10, csa-miRn8-3p, csa-novel-23, miRn6, PC-3p-318,270, PC-5p-100,383, PC-5p-274,347, csa-miRn6-3p, and PC-3p-45,338 with cleavage and/or translation inhibition. While *Csa2G354820* was only targeted by csa-miR-n01 with translation inhibition.

### Location detection of typical CsZFPs

**Fig. 4:**
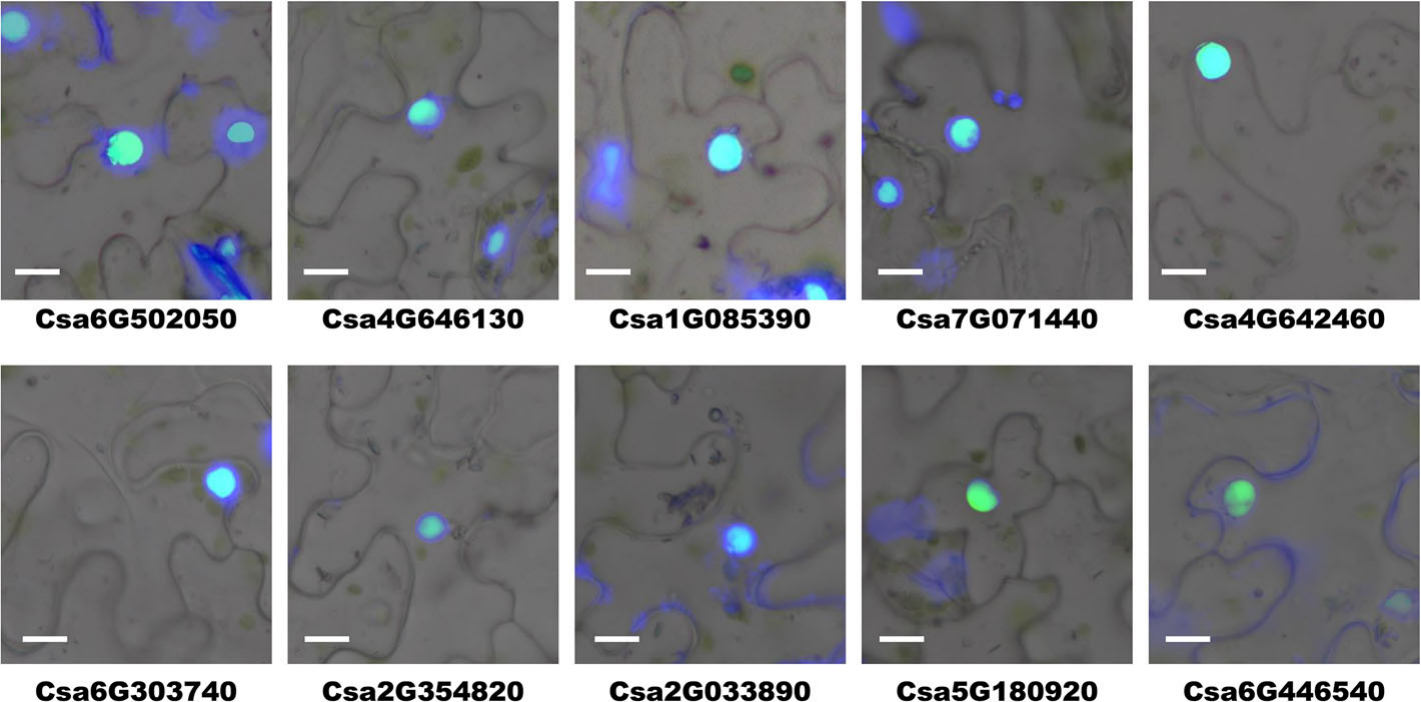
The subcellular location detection of typical CsZFPs. Images were merged by bright field, green fluorescence, and DAPI channels (Bar = 25 um). The photos were taken by Junliang Yin

The in silico prediction indicated that most CsZFPs located at the nucleus. In order to verify this result and reveal CsZFPs function patterns, 10 typical CsZFPs from different groups were selected to do the subcellular localization analysis. As shown in Fig. 4, all the tested CsZFPs, including Cas6G502050, Cas6G303740, Cas4G646130, Cas4G642460, Cas1G085390, Cas7G071440, Cas2G354820, Cas2G033890, Cas5G180920, and Csa6G446540, located at the nucleus, which were consistent with the prediction, suggesting the CsZFPs might contribute their biological roles in cucumber nucleus [31]. However, besides activate or suppress gene transcription in nucleus, zinc finger domain containing proteins can also involve in the regulation of protein-protein interaction, histone methylation, double stand RNA binding [32]. Thus, the precise functions of these CsZFPs need to be further experimentally explored.

### Expression profiling of CsZFP genes in cucumber different growth and developmental stages

**Fig. 5:**
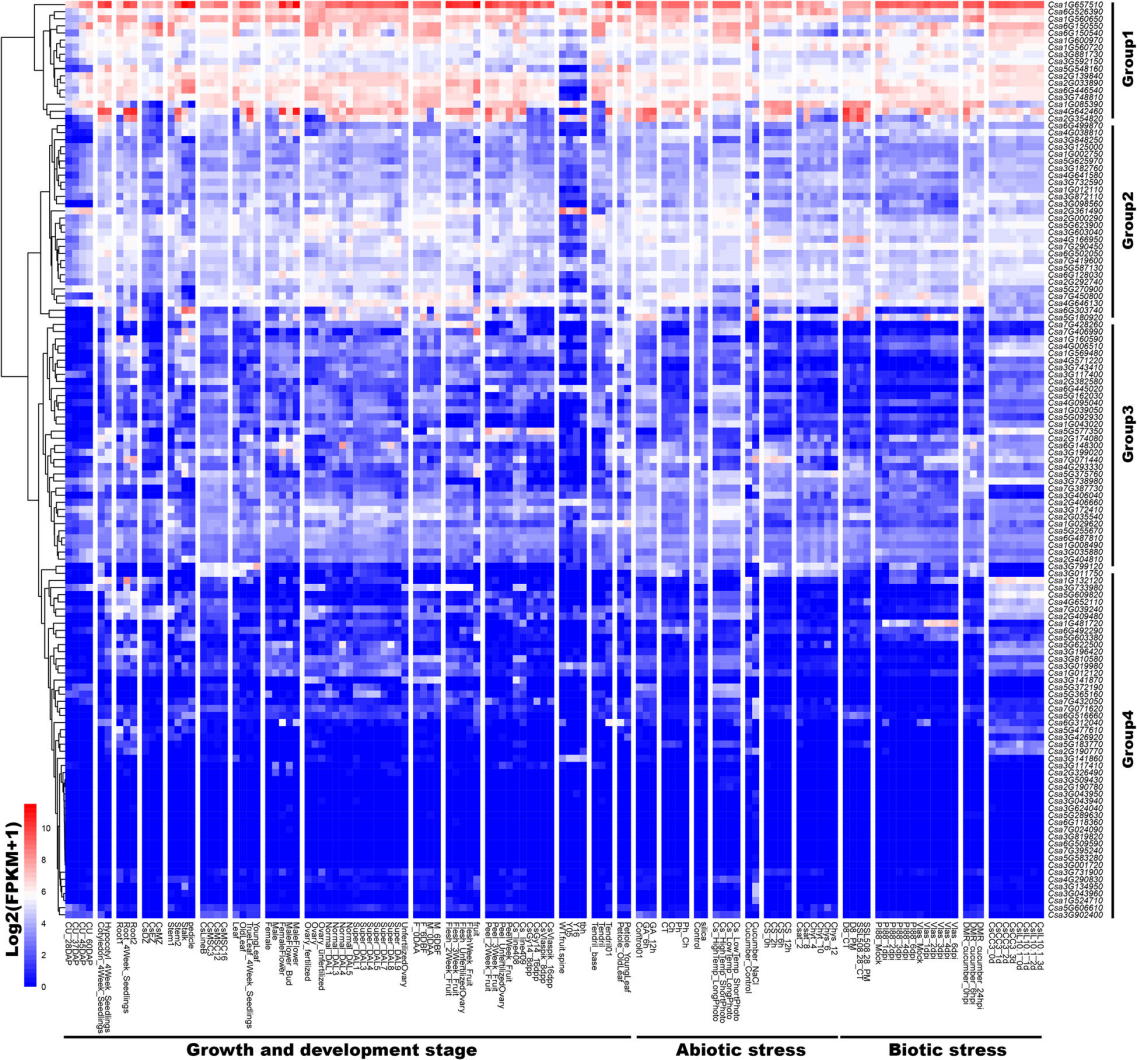
The RNA-seq analysis of different expression pattern of *CsZFPs* within heatmap pattern in response cucumber growth, development and biotic and abiotic stresses. The log2-transformed FPKM (Fragments Per Kilobase of transcript per Million fragments mapped) values of the dataset were used to do the expression analysis. The figure was drew by Junliang Yin

In attempt to detect the role of *CsZFPs* during the cucumber different growth and developmental stages, the expression levels of each *CsZFPs* were analyzed by the available RNA-seq transcriptomic data. According to the log2-transformed FPKM (fragments per kilobase of transcript per million fragments mapped) values of the dataset, the different expression pattern of *CsZFPs* were shown in heatmap figure (Fig. 5 and Additional file 1: Table S6). These *CsZFPs* expressed in different tissues, mainly including root, cotyledon, hypocotyl, stem, leaves, flower, seed. According to their expression levels, these *CsZFPs* could be clustered into four groups that the genes in group 4 show none or low expression level in all the tissues, but the expression level of genes in group 1 were much higher and significantly responsive to different treatments (Fig. 3). For instance, after 4 days of planting, the expression level of *CsZFPs* show different patterns in three root developmental regions, such as differentiation zone, elongation zone and meristematic zone. Among them, the expression levels of *Csa1G657510* and *Csa6G150550* were highly induced in the three parts, in contrast, 26 of *CsZFPs* showed none expression. While, two genes (*Csa1G132120* and *Csa5G548160*) were abundantly expressed in root differentiation zone, but were not in meristematic zone. In cotyledon and hypocotyl of 4 weeks seedlings, it is notable that *Csa1G657510* was significantly highly expressed. In addition, the expression levels of these genes were different in stem, stalk, and pedicle. Gene expression values for *Csa1G657510* were relatively higher in stem (maximum FPKM 626.52), in stalk (maximum FPKM 2958.85), and in pedicle (maximum FPKM 1661.78), respectively. Among the old and young leaves, the FPKM expression level of each *CsZFP* ranges from 0 (*Csa1G524710*) to 603.39 (*Csa1G657510*) and 0 (*Csa2G190780*) to 362.59 (*Csa1G657510*), respectively. In the fruit and fruit peels, *Csa6G303740* and *Csa7G406990* showed high expression levels in fruit, but not in fruit peels. All these results shown that the different expression of *CsZFPs* might function importantly and diversely in various tissues.

Except the detection of expression level of each *CsZFP* in different tissues, their functions were also analyzed during tissue developmental stages, such as different time points after pollination, fruit spine on different fruits. After pollination of 28, 37, 45, and 60 days, most of the *CsZFPs* showed increasing expression patterns with time development. Of them, the expression level of *Csa6G150550* increased significantly (15.41 to 335.51) from 28 to 60 days after pollination. While the *Csa7G450800* shown opposite expressing pattern that its expressing level decreased from day 28 (3.31) to day 60 (1.12). Moreover, the expression level of *CsZFPs* in fruit spine on fruits of 0.5 and 1.6 cm long showed distinctive expressing pattern. The expression levels of *Csa1G560650* and *Csa2G139840* showed none significant difference, but again most of them showed increasing patterns with the development of fruit.

### Expression analyses of the CsZFPs under biotic and abiotic stresses

To gain insights into the expression of *CsZFPs* in response to biotic and abiotic stresses, firstly the log2-transformed FPKM values of the RNA-seq transcriptomic dataset were mined as shown in Fig. 5. Within the gibberellin (GA) treatment, most of the *CsZFPs* shown none significant expressing changes in 12 h treatment, but the expression level of *Csa4G642460* increased strongly from 253.87 to 647, as well as other *CsZFPs* (*Csa4G166950* and *Csa7G450800*) which showed similar expression pattern in response to GA treatment. While, *Csa6G446540*, *Csa5G623900*, *Csa3G748810*, and *Csa3G098560* showed decrease patterns in their respective transcription levels. In response to salt stress, *Csa1G600970*, *Csa2G404810*, and *Csa7G419600* were highly induced. Moreover, in response to biotic stresses, most of the *CsZFPs* showed increase or decrease expressing patterns in 4 weeks old leaf infected with downy mildew, indicating these *CsZFPs* function differently in response to downy mildew. To sum up, the *CsZFPs* from group 1, 2, and 3 likely play more important roles during cucumber growth and development, and responses to biotic and abiotic stresses.

**Fig. 6:**
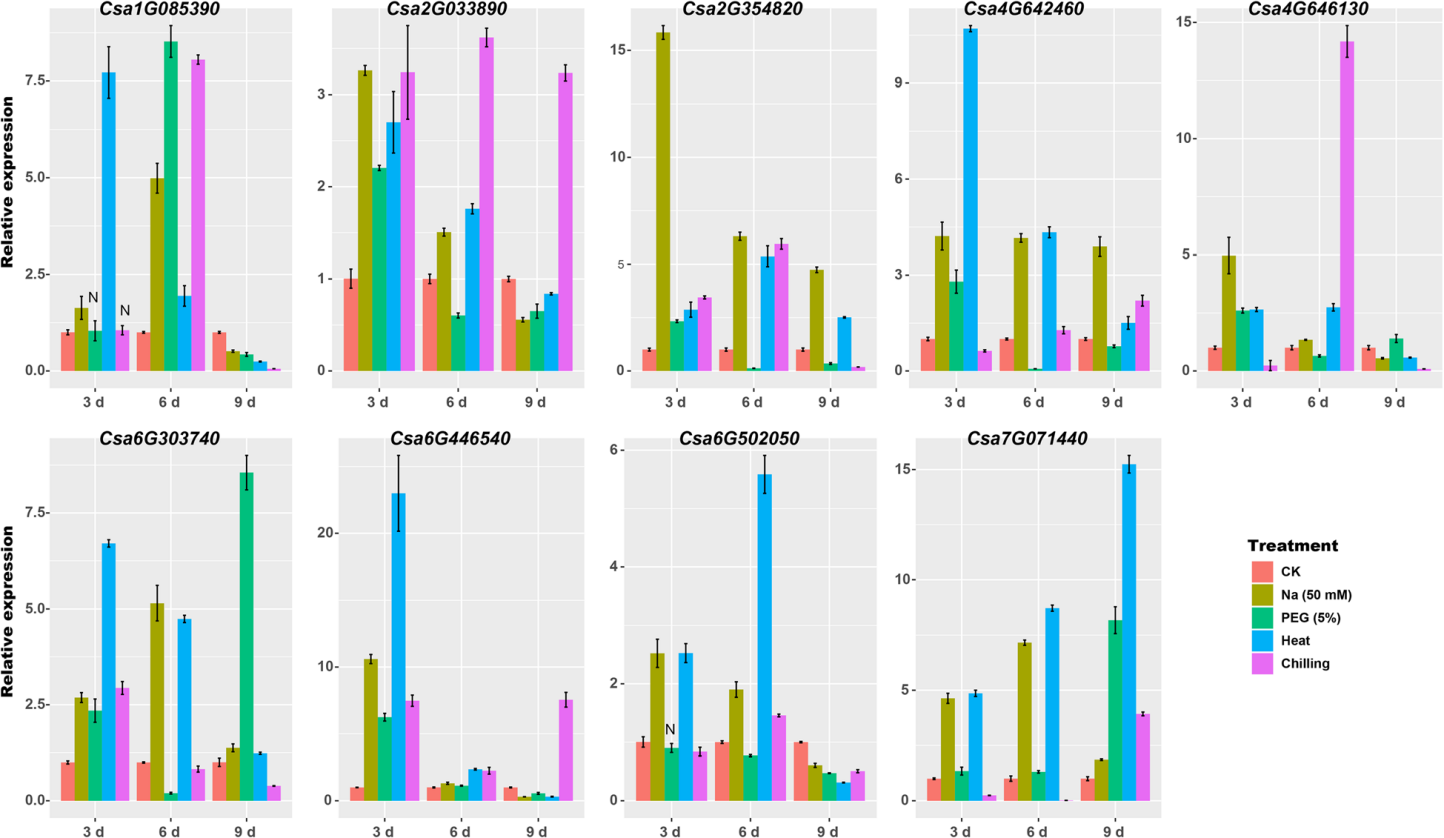
Relative expression profiles of *CsZFPs* in response to salt, drought, heat, and cold stresses. The relative expression levels of the representative members of *CsZFPs* in three independent replications and the error bars represent standard deviation (SD). N indicates none significant difference. The figure was drew by Junliang Yin

Furthermore, nine *CsZFPs*, which showed relatively higher expression levels and/or had orthologous been functional studied in other plants (e.g. *Arabidopsis*, rice) were selected to do the qRT-PCR analysis of their expression patterns in responding to salt, drought, heat, and cold stresses (Fig. 6). Seven *CsZFPs* were significantly induced in 3 d salt treatment and two *CsZFPs* (*Csa1G085390* and *Csa6G303740*) expressed highly within 6 d salt treatment comparing to the control. The most remarkable case is the gene *Csa2G354820* which was induced by more than 15-fold under salt treatment at 3 d. Then in the case of drought stress, the expression levels of *Csa1G085390*, *Csa6G303740*, and *Csa7G071440* culminated to the peak with 7.5-fold changes with 6 d, 9 d and 9 d drought treatment, respectively. While the others showed lower expression in response to drought stress. In response to heat stress, eight *CsZFPs* were up regulated with 3 d or 6 d treatment and then decrease to the lower expression level in 9 d treatment. The highest induction was recorded for *Csa6G446540* which showed > 20-fold up-regulation at 3 d comparing to the control. However, *Csa7G041440* was progressively inducted along with the time and reached to 15-fold at 9 d comparing to the control. In the case of cold stress, two *CsZFPs* (*Csa1G085390* and *Csa4G646130*) were significantly induced in 6 d treatment with 7.5 and 14-fold changes, the others were slightly induced. The results showed that all the nine *CsZFPs* were responsive to these four abiotic stresses.

### Csa4G642460 and Csa6G303740 are cell death inducer

**Fig. 7:**
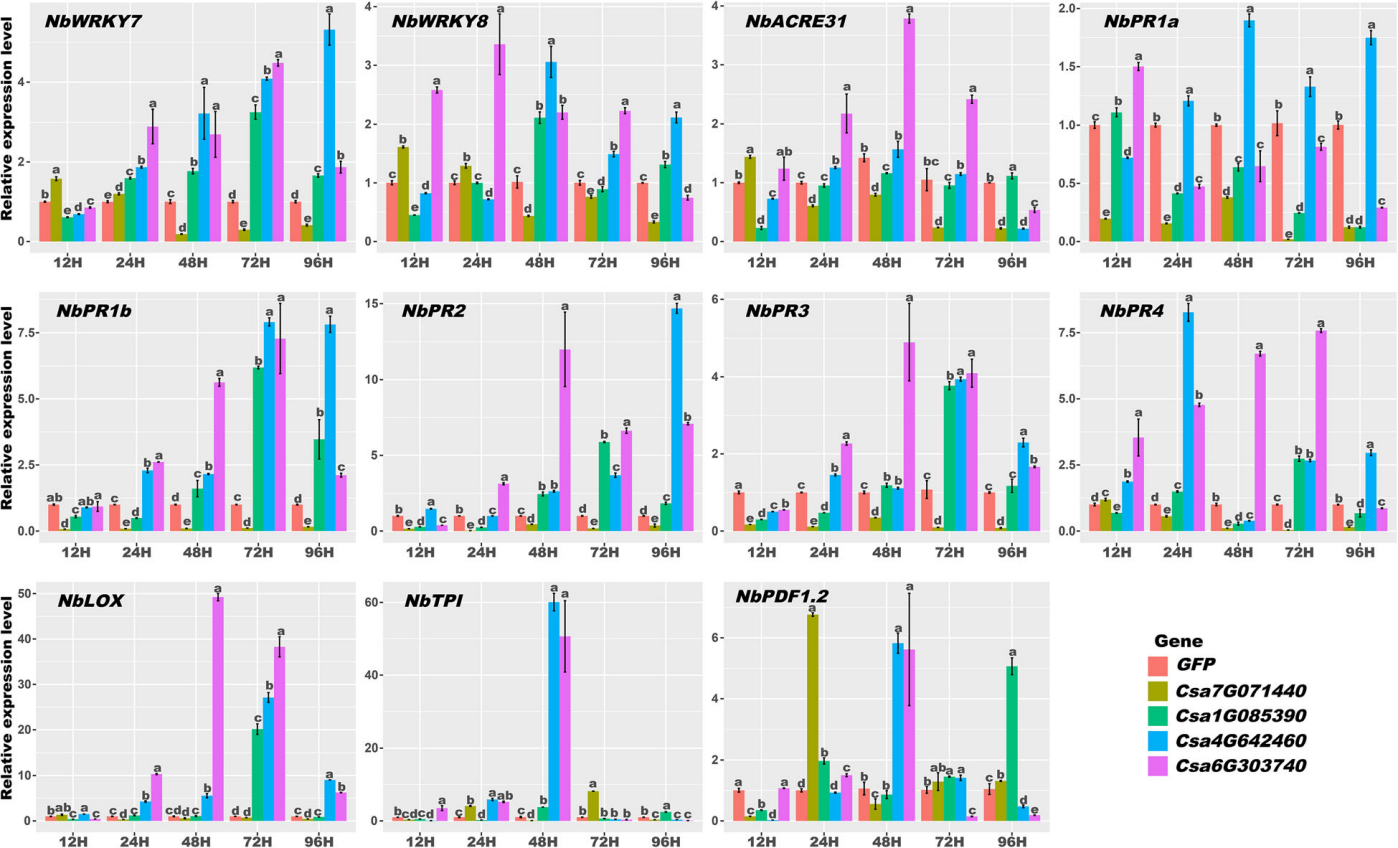
Cell death analyses. Those *N. benthamiana R* genes (*HSP90*, *SGT1*, *RAR2*, *MEK1*, *SIPK* and *WRKY3–2*) were transiently silenced by VIGS approach. Then **a***Csa4G642460* and **b***Csa6G303740* were transiently overexpressed in leaves of silencing plants. Whilst GFP, *Csa4G642460*, and *Csa6G303740* were parallel overexpressed in leaves of not silencing plants as control. Similar results were obtained from three independent replicates with 10 plants for each TRV construct. The photos were taken by Junliang Yin and the figure was drawn by Junliang Yin

After 5 days transient expression of *Csa4G642460* and *Csa6G303740* in *N. benthamiana* leaves, cell death could be observed in agro-infiltration corresponding regions (Fig. 7a and b). To reveal whether the cell death is *N. benthamiana* specific, *Csa4G642460* and *Csa6G303740* were transiently expressed in *Solanum lycopersicum*. It was observed that *Csa4G642460* and *Csa6G303740* are also cell death inducers in tomato leaves (Additional file 2: Fig. S2). Cell death usually depends on plant immune system and is the outcome of variable receptors and defense signal transduction pathways. In order to verify which signaling transduction pathways involved in *Csa4G642460* and *Csa6G303740* regulated cell death, a series of genes which are responsible for R (resistance) protein function, such as HSP90, SGT1, and RAR2, as well as the MAPK cascade genes including MEK1 and SIPK, and WRKY3–2 transcription factor were silenced one by one through virus-induced gene silencing (VIGS) method. As shown in Fig. 7a and bs, after silencing of these genes in *N. benthamiana*, the cell death inducer ability of *Csa4G642460* and *Csa6G303740* were not abolished. In summary, the *Csa4G642460* and *Csa6G303740* are cell death inducer and the inducing ability does not require above *R* genes among the signaling transduction pathways.

### Csa1G085390 promotes and Csa7G071440 represses pathogen colonization in N. benthamiana

**Fig. 8:**
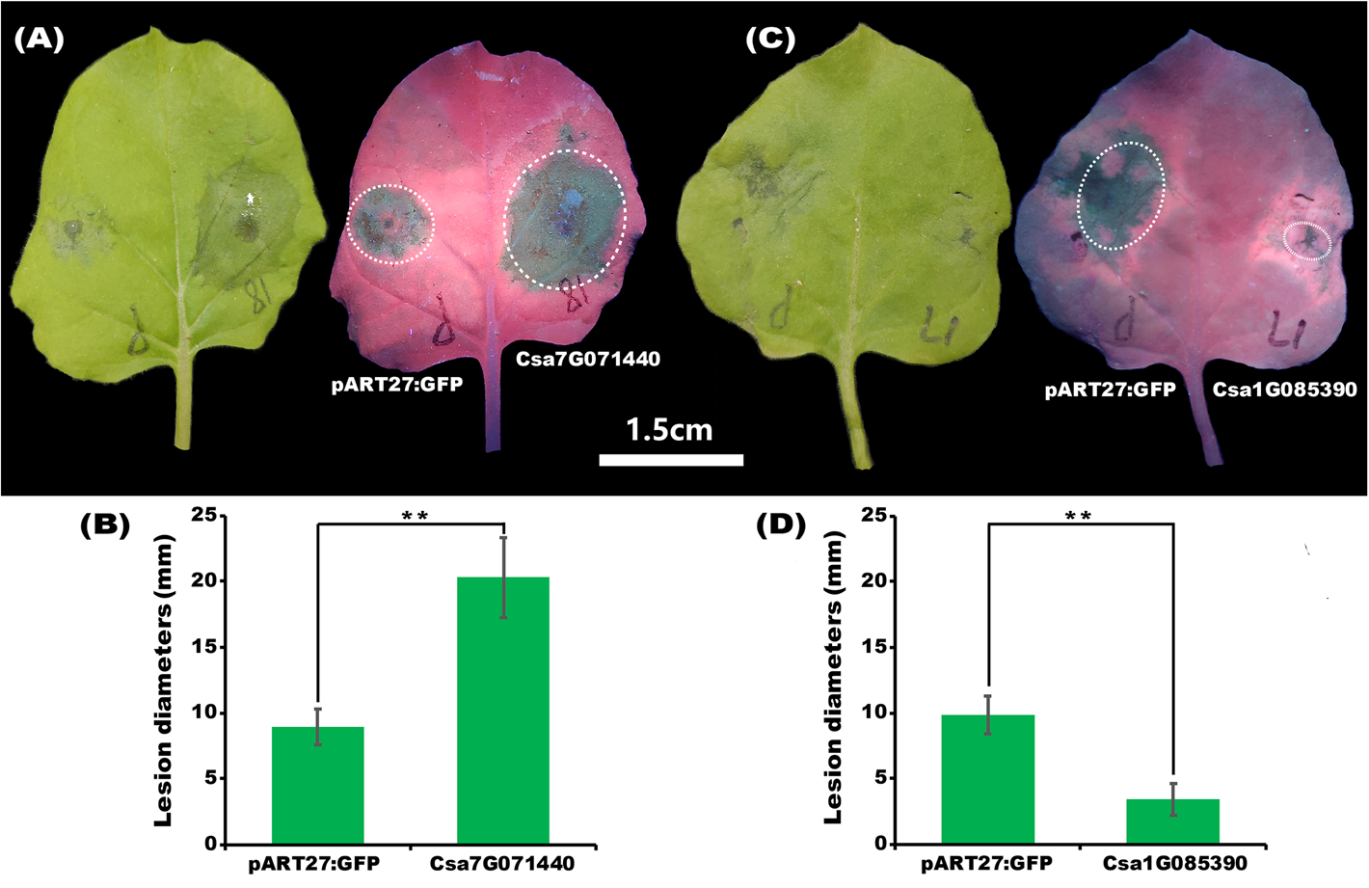
**a** Phenotype of transiently transgenic (*Csa1G085390* and *Csa7G071440*) and negative control (pART27:GFP) plants in response to pathogen attack. **b** Lesion diameter of necrosis in transgenic and control plants. Three independent replicates were observed and 30 leaves were used to do the quantification analysis. ** indicates significant differences at the level of 0.01. Scale bar: 1.5 cm. The photos were taken by Junliang Yin and the figure was drawn by Junliang Yin

Previous transient expression test showed that *Csa1G085390* and *Csa7G071440* are not cell death inducer. In order to further analyze the function of *Csa1G085390* and *Csa7G071440* in response to biotic stress. The pathogen of *N. benthamiana*, *Phytophthora infestans* (strain 88,069), was inoculated into tobacco leaves to check the phenotypes of these two transiently transgenic plants. As shown in Fig. 8a and c, the size of disease lesion in *Csa7G071440* transiently expressed plants was larger than that in the negative control plant. However, the opposite phenomenon was observed in *Csa1G085390* transient expression plants, indicating *Csa1G085390* and *Csa7G071440* function differently in response to pathogen attack. In addition, the leave lesion diameter statistical analysis supported above results (Fig. 8b and d) that the lesion diameter in *Csa7G071440* transiently expressed plants was significantly larger than that of negative control plants, while that in *Csa1G085390* transiently transgenic plants was significant smaller.

### Csa1G085390 and Csa7G071440 enhance salt and drought tolerance through initial induction of H_2_O_2_ in N. benthamiana

**Fig. 9:**
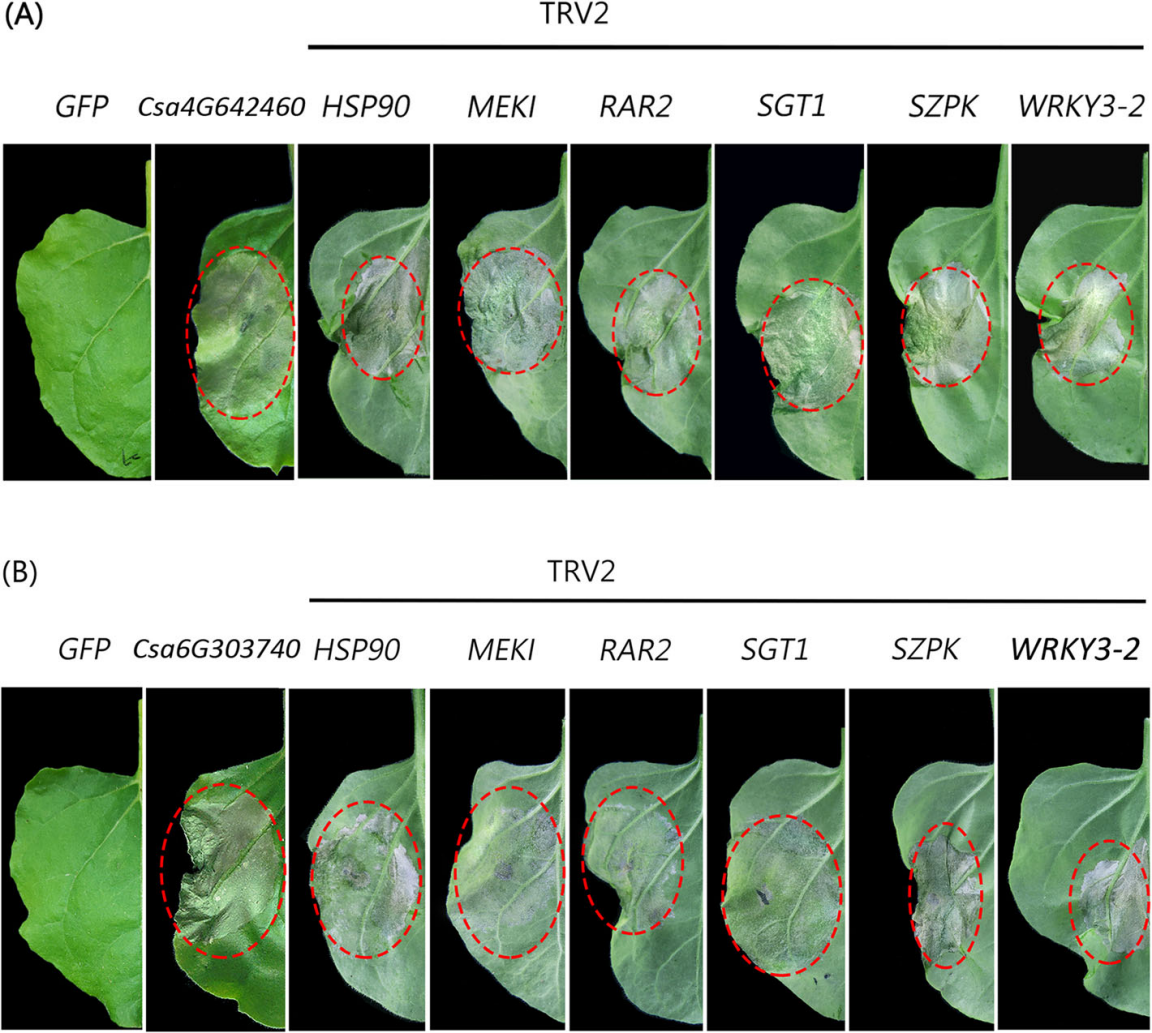
Phenotypic and physiological analysis of transiently transgenic (*Csa1G085390* and *Csa7G071440*) and negative control (pART27:GFP) plants under salt and drought stresses and normal condition (CK) after 4 h treatment. **a** Phenotypic comparison of transiently transgenic and negative control plants under salt and drought stresses. Corresponding water treatment was performed as negative control treatment (CK). Measurements of Water loss rate **b**, Hydrogen peroxide (H_2_O_2_) content **f** and malondialdehyde (MDA) **g** content in above treated leaves, respectively. Different letter indicates significant differences at the level of 0.05. **c** Stomata observation in transiently transgenic and negative control plants’ leaves. 30 leaves were observed for each plant and each treatment. **d** Quantification of stomatal apertures in the leaves of the transiently transgenic and control plants. Different letter indicates significant differences at the level of 0.05. **e** H_2_O_2_ detection by 3,3′-diaminobenzidine (DAB) staining in the leaves of the transiently transgenic and control plants . Scale bar: 5 cm. The photos were taken by Junliang Yin and the figure was drawn by Junliang Yin

*Csa1G085390* and *Csa7G071440* are the two typical *CsZFPs* which highly expressed in response to salt and drought stresses, suggesting they might play critical roles under these two stresses (Fig. 6). In order to verify this hypothesis, the performance of the transiently transgenic plants (*Csa1G085390* and *Csa7G071440)* was analyzed under salt and drought stresses. As shown in Fig. 9a, the detached leaves of *N. benthamiana* grew well without any stress treatment at the normal condition (CK) within 4 h treatment. However, with 75 mmol NaCl and 5% PEG treatments, the leaves in the negative control plants become wilt and rolling. Comparing to CK condition and negative control plants, the leaves in *Csa1G085390* transiently transgenic plants were healthy and did not rolling under drought stress than that in salt stress, but the *Csa7G071440* transiently transgenic leaves shown resistance to both stresses. Then water loss rate in the above plants were detected (Fig. 9b), the transiently transgenic leaves exhibited decrease of water loss rate comparing with the negative control under CK condition. However, with NaCl or PEG treatments, water loss rate decreased significantly within the negative control but not in the transiently transgenic leaves, indicating the higher water loss rate under these two stresses in transiently transgenic plants which have more ability to absorb water to improve their stress resistance. As the difference in water loss rate in the leaves of transiently transgenic and control plants could be correlated with the difference in leaf stomatal aperture, thus the status of stomatal aperture was analyzed. Under normal conditions, the transiently transgenic plants exhibited a marked decrease in leaf stomatal openings (Fig. 9c). In addition, the decrescent of stomatal apertures in negative control plant was higher than that of transiently transgenic plants which was consistence with the lower water loss rate trends under both stresses (Fig. 9d). Moreover, the contents of H_2_O_2_ were evaluated by DAB staining and accurate measurements (Fig. 9e and f), respectively. Under normal conditions, the brown (indicating H_2_O_2_) areas were significantly increased within transiently transgenic plants. Under both stresses, the brown color became deeper in all plants, but the increasement in transiently transgenic plants was lower than that in negative control which is consistence with the accurate contents of H_2_O_2_ in Fig. 9e. Furthermore, the MDA contents were also measured and the lower contents of MDA in transiently transgenic plants were observed than that in negative control under both stresses (Fig. 9g). All the results indicated that the transient overexpression of *Csa1G085390* and *Csa7G071440* could improve both stress resistance with the initial accumulation of H_2_O_2_ through triggering the early self-defense system.

### Expression analysis of pathogenesis-related (PR) genes in Csa1G085390, Csa7G071440, Csa4G642460, and Csa6G303740 transiently transgenic plants

**Fig. 10:**
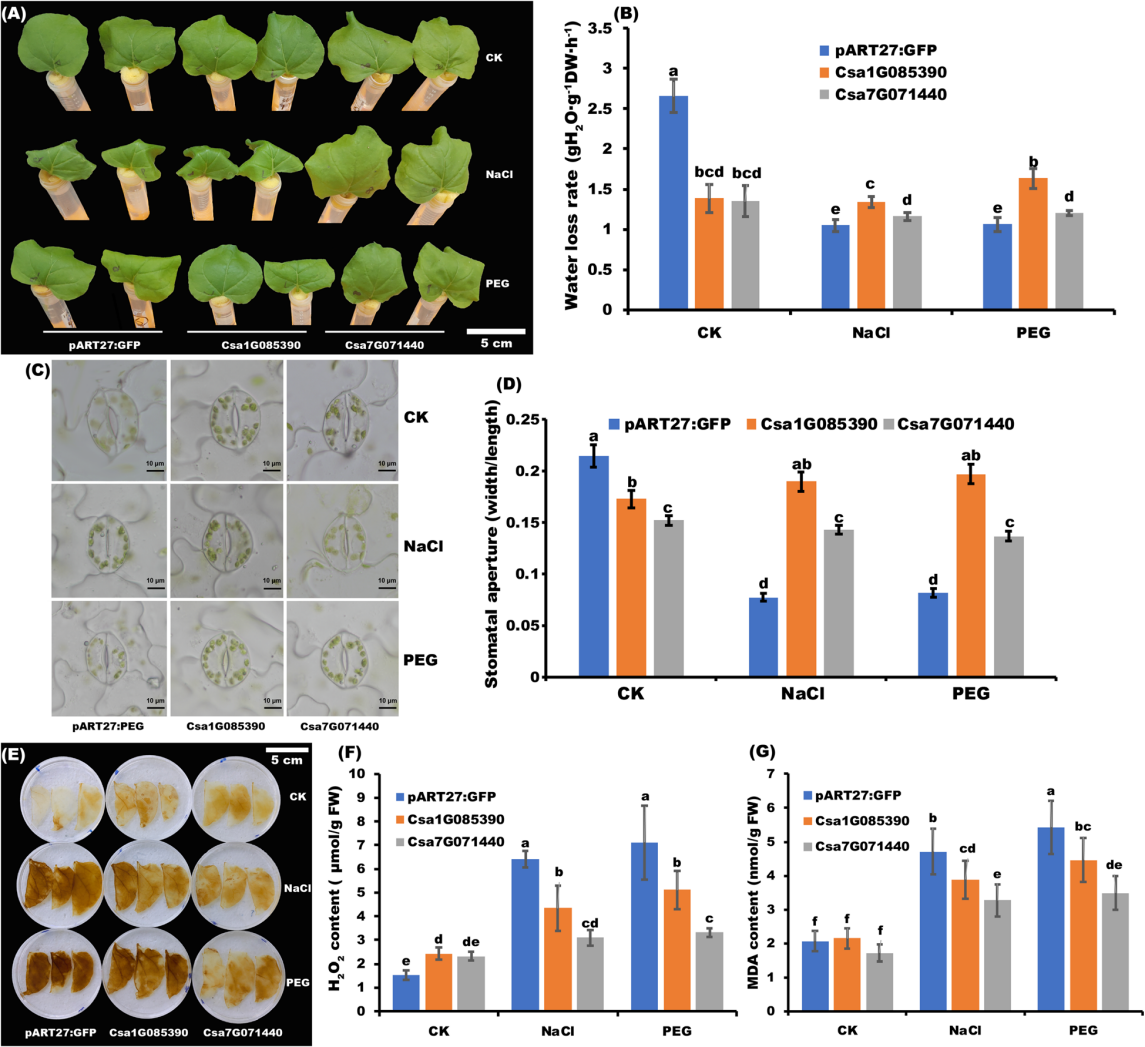
Relative expression profiles of pathogen response genes in transient overexpression of *Csa1G085390*, *Csa7G071440*, *Csa4G646130* and *Csa6G303740* plants after 12, 24, 48, 72 and 96 h of *P. infestans* infection. Each bar represents the average of three replicates, and error bar represents standard deviation (SD). Different letter indicates significant differences at the level of 0.05. The photos were taken by Junliang Yin and the figure was drawn by Junliang Yin

As discussed above, the *CsZFPs* could be induced by abiotic and abiotic stresses. Thus, four *CsZFPs*, including *Csa1G085390*, *Csa7G071440*, *Csa4G642460*, and *Csa6G303740*, were transiently overexpressed into *N. benthamiana* leaves. Then three PTI related genes (*NbWRKY7*, *NbWRKY8* and *NbACRE31*), three SA response genes (*NbPR1a*, *NbPR1b* and *NbPR2*), and five JA-dependent immunity genes (*NbPR3*, *NbPR4*, *NbLOX*, *NbTPI* and *NbPDF1.2*) were selected to do the expression analysis in these transgenic plants. As shown in Fig. 10, most of the genes in *Csa7G071440* transiently transgenic plant were down regulated within 12 h, such as *NbWRKY7*, *NbWRKY8*, *NbPR1a*, *NbPR1b*, *NbPR2* and *NbPDF1.2*, which demonstrated overexpression of *Csa7G071440* could decrease the PR genes expression to suppress the plant defense. This might be the reason why the larger disease lesion was observed in *Csa7G071440* transgenic plant after pathogen infection as shown in Fig. 8a and b. However, the expression levels of these genes were up regulated in *Csa1G085390* transiently transgenic plant at specific time point. For example, *NbWRKY7* was highly induced at 24 to 48 h. *NbTPI* was up regulated at 48 h time point. This could give the explanation that *Csa1G085390* transiently transgenic plant was resistance to pathogen attack (Fig. 8b and c). Moreover, the expression levels of these genes were mostly up regulated in other two transiently transgenic plants (*Csa4G642460* and *Csa6G303740*) from 12 h to 96 h. As exemplified, the *NbWRKY 8* was highly induced in 12 h and the *PR* genes, such as *NbPR1b*, *NbPR2*, *NbPR3,* were significantly up regulated from 24 h to 72 h in *Csa6G303740* transgenic plants. In addition, the same expression changes of *NbWRKYs* and *NbPRs* were observed in *Csa4G642460* transgenic plants. These results could demonstrate the *Csa4G642460* and *Csa6G303740* genes could trigger the expression of *PR* genes to activate the hypersensitive response (leave cell death as shown in Fig. 7).

## Discussion

The C2H2-type of zinc finger proteins that widely existed in eukaryotic kingdom play critical roles in many biological processes, such as hormone signaling, DNA or RNA binding, and stress responses [9, 33]. In addition, the genome wide identification of C2H2-type ZFPs has been conducted in several plant system, whereas not in cucumber. Thus, in order to get insights into the function of C2H2-type ZFPs in cucumber, the comprehensive analysis of *CsZFPs* was performed in the current study. Finally, 129 full-length *CsZFPs* were identified (Table 1), which could be classified into four groups according to the variation of the plant-specific conserved amino acid sequence “QALGGH” and distances between metal ligands within C2H2-ZF domains (Fig. 2). The principle classification of C2H2-ZFPs based on the number and structural ZF domains has been firstly studied in yeast and *Arabidopsis* [5, 34]. In different plant species, the modification of the conserved motif “QALGGH” was widely used to do the classification [35]. In cucumber, majority of CsZFPs contain one or two C2H2 domains with the QALGGH motif were identified as Cs1Q and Cs2Q subgroups (Additional file 1: Table S2). The same typical group has been identified in *Brassica rapa* L., which suggested that these proteins may be involved in plant-specific life processing [12, 31]. Moreover, the other three groups which do not contain this typical ZF domain have been identified, implies the functional diversity of these C2H2-ZFPs in plant growth and development. With the location analysis, most of the CsZFPs were predicted to be located in the nucleus and these have been confirmed by the subcellular location analysis, suggesting that they indeed function as transcription factors in nucleus, but the Csa7G406990 which comes from Cs1Q group located in cell wall and Csa2G292740 which belongs to Cs1C group located in endoplasmic reticulum (Table 1 and Additional file 1: Table S2), indicating the CsZFPs members in the same phylogenetic group did not necessarily correspond to the same subcellular localization. Moreover, except the DNA or RNA binding function of C2H2-ZFPs in the nucleus [31], the members of CsZFPs which did not locate in the nucleus might have other special function and play important roles in signal transduction.

The structural and physicochemical properties of a gene family analysis could indicate the diversity of each member during the evolutionary process [36]. Our results demonstrated a wide range of variations in molecular weight, theoretical pI values, chromosome location and exon-intron number, which elucidates the evolutionary changes occurred in cucumber likely via gene duplications and/or integration into genomic regions after reverse transcription [9]. Duplication modes of genes, including segmental and tandem duplication, are considered to be characteristic features and primary driving forces of the evolution of genomes [37]. Tandem duplicates are defined as paralogous genes that are adjacent to each other on a chromosome [38]. In the current study, gene duplication analysis revealed that no tandem and segmental duplication took place which was consistent with the fact that cucumber genome was absence of recent whole-genome duplication events and tandem duplication [39, 40]. The smaller number of *CsZFP* genes compared with *Arabidopsis*, rice and rape, may also be attributed by the absence of recent duplication events in cucumber genome.

CsZFPs are important kind of transcription factors in the regulation of plant growth and development, hormones metabolisms and stress responses. While *cis*-elements existed in the promoter region play key roles in the transcriptional level of genes in response to multiple stimuli [41]. Our results identified several *cis*-regulatory elements in related to plant growth and development, phytohormone, biotic and abiotic stresses, including TATA-box, ABRE, G-box, in corresponding promoter region of *CsZFPs* (Fig. 3 and Additional file 1: Table S3). All these *cis*-regulating elements have been identified in the study of *Arabidopsis* [42, 43]. Thus, the prediction of extensive *cis*-regulatory elements in the promoter regions of *CsZFPs* suggested that the *CsZFPs* might play essential roles in response to various stresses and phytohormone regulation. In addition to the function of *cis*-regulating elements, miRNAs also play essential roles in the development of plant growth, stresses and other morphological processes [44]. For instance, the tae-miR9674a-5p, tae-miR9781 and tae-miR9655-3p which were identified in wheat function importantly in various metabolisms and biological processes [7]. In cucumber, the number of miRNAs target to corresponding *CsZFPs* transcripts ranges from 1 to 20 (Fig. 3 and Additional file 1: Table S5), indicating that different miRNAs could regulate the translation process of *CsZFPs* during multiple physiological and stress-induced cellular responses which were consistence with the study in durum wheat [7].

All the prediction of *cis*-regulating elements and miRNAs in response to *CsZFPs* suggested they could be involved in cucumber growth and development, and various stresses. Furthermore, multiple studies showed that most of C2H2-ZFPs function as transcription factors are widely employed in plant growth and development [45, 46]. In the current study, with the publicly available RNA-seq data analysis, the expression patterns of *CsZFPs* demonstrated that these genes are involved in tissue and organ development, especially root and floral development (Fig. 5), illustrating the important function of this gene family in cucumber growth and development. The same conclusion was got from the study of *Brassica* which showed that the high expression level of most of *B. rapa* C2H2-ZFP genes was observed in at least one of tested tissues and furthermore one hundred of them were significantly expressed in six tissues [12]. These genes might function importantly in tissue growth and development of cucumber species and deserved to be further studied at their molecular levels. For example, *JcZFP8*, a C2H2 zinc finger protein gene from *Jatropha curcas* L., participates the trichome development in transgenic tobacco [47]. The C2H2-typezinc finger protein named *ZINC FINGER PROTEIN 1* (*AtZP1*) negatively regulates *Arabidopsis thaliana* root hair initiation and elongation [48].

In addition to the function of *CsZFPs* in cucumber growth and development, transcription profiling analyses have demonstrated that the transcript level of many C2H2-type zinc finger proteins is elevated under different abiotic stress conditions such as low temperature, salt, drought, osmotic stress, and oxidative stress [8]. For example, *ZAT12* has been reported to be involved in multiple abiotic stresses in *Arabidopsis* [49]. In addition, *ZAT18* was found to be a positive regulator in response to drought stress in *Arabidopsis* [19]. In banana, two C2H2-ZFPs, named as *MaC2H2–1* and *MaC2H2–2*, were identified as transcriptional repressors to repress the expression of ethylene biosynthetic genes in fruit ripening [50]. In cucumber, a C2H2 zinc finger transcription factor, named as *Zat12* which could be up-regulated under melatonin treatment to alleviate chilling stress by modulation of polyamine and abscisic acid metabolism [51]. Moreover, *AtAZF2* play important roles in the regulation of ABA-repressive and auxin-inducible genes under abiotic stress conditions [20, 52]. The expression analysis of three other TFIIIA zinc finger proteins, *AZF1*, *AZF2* and *AZF3* show that *AZF1* and *AZF3* were induced under cold stress but not in ABA treatment [53]. Interestingly, in our study, the orthologous gene of *AtAZF2* in cucumber is *Csa4G642460* which was up regulated in response to drought, salt and heat stresses, but not response to cold stress (Fig. 6), which was consistence with above results and demonstrated they function essentially under these three abiotic stresses. Furthermore, *ZFP245*, a C2H2-type zinc finger protein, is highly induced under drought stress and overexpression of *ZFP245* increased the tolerance of rice to drought stress [54–56]. While *Csa6G303740*, the orthologous gene of *ZFP245*, was significantly up regulated in response to drought stress as well as heat stress, indicating it could involve in the regulation of drought and heat stresses in cucumber.

In the current study, nine *CsZFP* genes are significantly induced by drought, cold, heat, and salt stresses, implying the involvement of these genes in response to abiotic stresses (Fig. 6). As an important ROS, H_2_O_2_ functions as a signal molecule in plant responses to abiotic stress [28]. Furthermore, transient overexpression of *Csa1G085390* and *Csa7G071440* into *N. benthamiana* could enhance salt and drought stresses through the initial induction of H_2_O_2_ (Fig. 9). ROS and redox cues could activate stress acclimation process by retrograde signaling to regulate metabolic fluxes [57]. Here, the initial accumulations of H_2_O_2_ in these transgenic plants function as signaling molecule to activate the defense mechanism against drought and salt stresses. Besides, *Csa1G085390* promotes and *Csa7G071440* represses pathogen colonization in *N*. *benthamiana*, suggesting that *Csa1G085390* could weaken plant defensive immune responses while *Csa7G071440* could strengthen the responses. Considering the fact that these two genes are not cell death inducer, they may not involve in the plant hypersensitive defense pathways. Moreover, the exact defense pathway mediated by these two genes are still unclear, which need to be further studied.

*Csa4G642460* and *Csa6G303740* could induce cell death in their overexpression plants, but the cell death could not be rescued by slicing *HSP90*, *SGT1*, *RAR2*, *MEK1*, *SIPK* and *WRKY3–2* genes (Fig. 7), indicating there must be other key regulators involved in this signaling transduction. Moreover, the PR genes was highly induced in these two transgenic plants which might illustrating the PR genes might be one potential candidates during *Csa4G642460* and *Csa6G303740* induced hypersensitive response Since cell death inducer usually play positive roles upon pathogen infection, *Csa4G642460* and *Csa6G303740* may also play roles in plant resistance to pathogen. However, their roles in pathogen resistance need to be further tested in cucumber. It is interesting that Csa6G303740 was also induced by salt stress, suggesting that it may also participate in salt response of cucumber. In the future study, the stable transformation of these two genes into cucumber in response to biotic and abiotic stresses needs to be further analyzed and confirming the molecular mechanism of *CsZFPs* under these stresses.

## Conclusion

In the current study, 129 full-length *CsZFPs* were identified, which could be classified into nine groups according to the phylogenetic analysis. The 129 *CsZFPs* unequally distributed on 7 chromosomes and no tandem duplication events took place during the evolutionary process. Promoter *cis*-element analysis showed that the CsZFPs might involve in the regulation of phytohormone or abiotic stress response, and different *CsZFPs* were regulated by several miRNAs. In addition, subcellular localization analysis indicated that most of the CsZFPs located in the nucleus and the RNA-seq analysis of *CsZFPs* demonstrated that these genes are involved in tissue and organ development. The expression profiling of *CsZFP*s by qRT-PCR analysis in response to abiotic stresses indicated that all the nine typical *CsZFPs* are significantly involved in drought, cold, heat and salt stresses. Furthermore, the transient overexpression of *Csa1G085390* and *Csa7G071440* into *N. benthamiana* plant revealed that they could decrease and induce leave necrosis in response to pathogen attack, respectively, and they could enhance salt and drought stresses through the initial induction of H_2_O_2_. In addition, *Csa4G642460* and *Csa6G303740* are cell death inducers in tobacco and tomato.

## Methods

### Identification of ZFP genes in cucumber

To identify all the *ZFP* genes in cucumber, two searching methods were performed. First, the protein sequences of cucumber (cucumber_ChineseLong_v2_pep.fa) were downloaded from the Cucurbit Genomics Database (http://cucurbitgenomics.org/organism/2). Then, the ZFP protein sequences of the *Arabidopsis*, rice, and maize were used as queries to identify the potential candidates by local BLASTP searching with a cutoff e-value 1e-10. Second, the keywords “zinc finger” were used as queries to search the Cucurbit Genomics Database (http://cucurbitgenomics.org/search/genome/2). Finally, all the retrieved non-redundant sequences were submitted to InterProScan (http://www.ebi.ac.uk/interpro/) and smart (http://smart.embl-heidelberg.de/) to assess the presences of C2H2 domains (IPR000690, IPR003604, IPR013085, IPR032553, IPR034736, IPR013087, IPR019406, IPR014898).

### Protein characterization, amino acid properties, chromosomal localization, gene structure and duplication analysis

Gene locus, chromosomal location, and the exon-intron structure of *CsZFP* genes were extracted from the genome gff3 annotation file. The approximate positions of *CsZFPs* were located to 7 chromosomes using software Mapinspect [36]. Gene duplications were classified into tandem duplication and segmental duplication events. Tandem duplication was determined according to Fang et al. [58]. The protein identification and analysis tools in ExPASy Server10 (https://prosite.expasy.org/) were used to predict length, molecular weight (MW), theoretical isoelectric point, instability index, amino acid composition, and atomic composition of CsZFPs.

### Multiple sequence alignment and classification of cucumber C2H2-ZFPs into groups

The multiple sequence alignments of the full-length protein sequences were performed by using ClustalW2 (v2.1) with default parameters. An un-rooted phylogenetic tree was constructed using MEGA7 package with the neighbor-joining method based on LG model, and bootstrapping was performed 1000 times [59]. The phylogenetic tree was illustrated using Interactive Tree of Life (IToL, v3.2.317, http://itol.embl.de). The conserved motifs of CsZFPs were identified using MEME motif search tool (http://memesuite.org/tools/meme) [60]. Default parameters were used in this study, except that the maximum number of motifs was set to 20. And the motif patterns were drawn by TBtools software (https://github.com/CJ-Chen/TBtools). Furthermore, SMART database [61] was used to analyze 129 *CsZFPs* manually to search the numbers, sequences of C2H2-ZF domain, and the space length between C2H2-ZF domains. According to the methods described in soybean and *Populus trichocarpa* [12, 13], the C2H2-ZF domains in CsZFPs were classified into five main types. Then, based on the types and numbers of C2H2-ZF domains, CsZFPs were further classified into nine groups. The detailed information was showed in results.

### Analysis of microRNA target sites and cis-acting regulatory elements

The *Cucumissativus* miRNA sequences were obtained from the miRBase database at http://mirbase.org/ [62] and the mature sequences of cucumber miRNAs were collected from the previously reported publications [26, 63]. To detect potential miRNA target sites within the *CsZFP* genes, the obtained miRNAs were analyzed with the psRNATarget server (http://plantgrn.noble.org/psRNATarget/) [64]. The up-stream 1500 bp DNA sequences of *CsZFP* genes were used to do the *cis*-acting regulatory elements analysis by PlantCARE database (http://bioinformatics.psb.ugent.be/webtools/plantcare/html/) [65].

### RNA-seq data analysis of CsZFP genes

To analyze the expression profiles of *CsZFP* genes, we collected the expression level of each *CsZFP* represented by FPKM values (Fragments Per Kilobase of transcript per Million fragments mapped values) from the Cucurbit Genomics Database (http://cucurbitgenomics.org/organism/2). The heatmap of cucumber *CsZFP* genes were generated using R package “pheatmap”.

### Plant materials and treatments

Cucumber (*Cucumis sativus* L. cv. JinYou 1, Xintiandi Co., Yangling, Shannxi, China) seeds were rinsed thoroughly in distilled water and germinated on moist gauze in an incubator at 28 °C in dark for 2 days [66]. The germinated seeds were sown in a mixed substrate (peat: vermiculite: perlite = 2:1:1) in an artificial growth chamber with an average of 12 h/12 h day/night light. The temperature were set to 28 °C/18 °C day/night. At two-leaf stage, the uniform seedlings were transferred to plastic containers filled with 15 L of 1/4 strength of Hoagland nutrient solution. Three days later, the strength of Hoagland solution was increased to 1/2. Seven days after transplanting, seedlings were subjected to four experimental groups: (i) Control, seedlings were incubated in the chamber at 28 °C/18 °C day/night temperature; (ii) Salt stress, 75 mM sodium chloride (NaCl) was added to the nutrient solution; (iii) Heat stress, seedlings were treated with 40 °C/32 °C day/night temperature in another identical growth chamber; (iv) Chilling stress, seedlings were exposed to 18 °C/5 °C day/ night temperature for chilling treatment [67]. The roots were collected after 3, 6, and 9 days of treatment respectively. The solution pH was maintained at 6.0 using 0.2 M H_2_SO_4_ or 1 M KOH. All sampled materials were harvested to be frozen with liquid nitrogen and stored at − 80 °C prior to RNA extraction.

### RNA extraction and qRT-PCR analysis

Total RNA extraction from the roots of cucumber and following cDNA synthesis were performed according to manufacturer’s instructions (Invitrogen, Carlsbad, CA) as described by Yin et al. (2018) [68]. The qRT-PCR was carried out on a CFX 96 Real-Time PCR system (Bio-Rad) using SYBR Green Master Mix (Vazyme, Nanjing, China) according to manufacturer’s protocols. The 20 μL reaction system contained 10 μL of 2 × SYBR Premix ExTaq™, 0.4 μL each of 10 μM primers, 1 μL diluted cDNA and 8.2 μL ddH_2_O. The thermal profile was pre-incubation for 3 min at 94 °C, followed by 40 cycles of 5 s at 94 °C, 15 s at 55 ~ 63 °C and 15 s at 72 °C. Relative expression levels of *CsZFPs* were calculated with the 2^-ΔΔCt^ method [69]. The primer sequences of *CsZFPs* and reference gene for qRT-PCR were shown in Additional file 1: Table S7.

### Subcellular localization analysis of CsZFP proteins

To confirm the sub-cellular localization of *CsZFPs*, ten *CsZFPs* from different groups were selected to do the subcellular analysis. Briefly, full lengths of *CsZFPs* were amplified by Phanta HS Master Mix (Vazyme, Nanjing, China). Then the PCR products were inserted into the *Xho*I digested vector pART27:GFP (NEB, Beijing, China) by using ClonExpress II One Step Cloning Kit (Vazyme, Nanjing, China). The *CsZFP*:GFP fusion constructs were transformed into *Agrobacterium tumefaciens* strain GV3101 and the pART27:GFP transformation was used as negative control. Tobacco (*Nicotiana benthamiana*) leaves were used to do the transformation by infiltration method. Two days later, the injected leaves were placed on the glass slides and visualized through fluorescence microscopy (Olympus FV3000, Tokyo, Japan) with 488 nm exciting light wavelength.

### Transient overexpression of four typical CsZFPs into N. benthamiana in response to biotic and abiotic stresses

Four typical *CsZFPs* (*Csa1G085390, Csa7G071440, Csa4G642460* and *Csa6G303740*):GFP fusion vectors were transient transformed into *N. benthamiana* plants by *Agrobacterium tumefaciens* strain GV3101 whilst the pART27:GFP transformation was used as negative control. The agroinfiltration leaves were collected after 12, 24, 48, 72, and 96 h treatment and quickly frozen in liquid nitrogen and stored at − 80 °C for RNA extraction. Then the relative expression levels of pathogenesis-related (PR) genes were calculated with the 2^-ΔΔCt^ method and the primer sequences of these genes were shown in Additional file 1: Table S8. Furthermore, the phenotype of *Csa1G085390* and *Csa7G071440* transgenic plants were assessed in response to pathogen attack (*Phytophthora infestans*). Briefly, after 2 days of transient expression, the detached leaves were inoculated by zoospores of *P. infestans* (strain 88,069) [70]. After 5 days of inoculation, the lesion diameter was measured and visualized using a handheld long-wavelength UV lamp (Blak-Ray B-100AP, Ultraviolet Products) and figures were taken using the Carl Zeiss Imaging System.

After transient expression pART27:GFP, *Csa1G085390*:GFP, and *Csa7G071440*:GFP fusions in *N. benthamiana* for 2 days, leaves were excised from the plants and the phenotype of these leaves in response to drought (1% PEG) and salt (400 mM NaCl) stresses were evaluated. In addition, water loss rate was conducted as described by Zhu et al. [36]. Stomatal apertures were examined in the abaxial epidermis of leaves by Olympus BX51 (Shinjuku-ku, Tokyo, Japan) microscope after 4 h of PEG and salt treatment. The quantification of stomatal apertures was measured by ImageJ. Finally, H_2_O_2_ and MDA concentration were determined as described by Yin et al. [26]. Moreover, the H_2_O_2_ accumulation was detected by histochemical staining diaminobenzidine (DAB) method [71].

### VIGS assay in N. benthamiana

The cell death in *Csa4G642460* and *Csa6G303740* transgenic plants were observed after 5 days transformation. The same phenotype of *Csa4G642460* and *Csa6G303740* transiently expressed in *Solanum lycopersicum* leaves were observed after 6 days transformation. Cell death inducing signaling transduction pathway related genes (*HSP90*, *SGT1*, *RAR2*, *MEK1*, *SIPK*, and *WRKY3–2*) were amplified and constructed to pTRV2 vector [72], which was mixed with pTRV1 in equal ratios to a final OD_600_ of 0.25. The pTRV2:GFP was used as a control. These constructed vectors were transformed into the lower leaf of four-leaf stage *N. benthamiana* by *Agrobacterium tumefaciens* strain GV3101, and 3 weeks later, the constructed vectors of *Csa4G642460*:GFP, and *Csa6G303740*:GFP were transiently expressed in the upper leaves. The degree of cell death was analyzed in the upper leaves after 5 days transformation.

## Supplementary information

**Additional file 1 Table S1.** The types and sub-types of C2H2-ZF domains and their characteristics. **Table S2.** Detailed information of C2H2 motifs in the classified C2H2-ZFPs. **Table S3.** The *cis*-regulatory elements analysis of the promoter region of *CsZFPs*. **Table S4.** The detailed information of *cis*-regulatory elements in the promoter region of *CsZFPs*. **Table S5.** The detailed information of miRNAs targeted to *CsZFPs*. **Table S6.** RNA-seq analysis of *CsZFP* genes in cucumber different growth, developmental stages and in response to biotic and abiotic stresses. **Table S7.** The primer sequences of *CsZFP* genes for qRT-PCR. **Table S8.** The primer sequences of pathogen response genes for qRT-PCR.

**Additional file 2 Figure S1.** The motif sequences and the conserved residuals in the motifs. **Figure S2.** Cell death analysis in tomato leaves. *pART27:GFP*, *Csa4G642460* and *Csa6G303740* were transiently expressed in *Solanum lycopersicum*.

## Data Availability

The genome data and sequences and expression profiles of *CsZFP* genes used in the current study are available in the Cucurbit Genomics Database (http://cucurbitgenomics.org/search/genome/2). All data generated or analyzed during this study are included in this published article and its Additional files. The datasets generated and analyzed during the current study are available from the corresponding author on reasonable request.

## Abbreviations

ZFPs: Zinc-finger proteins
CsZFPs: C2H2 ZFPs in cucumber
TFs: Transcription factor
Cys: Cysteine
His: Histidine
ABA: Abscisic acid
NaCl: Sodium chloride
AtZP1: Zinc Finger Protein 1
