# Supplementary material for: Genome-wide characterization of the C2H2 zinc-finger genes in Cucumis sativus and functional analyses of four CsZFPs in response to stresses
Source: BMC Plant Biol. 2020 Jul 29;20:359. doi: 10.1186/s12870-020-02575-1 (PMC7392682; doi:10.1186/s12870-020-02575-1)
Supplement: Supplementary file 2 — Additional file 2 Figure S1. The motif sequences and the conserved residuals in the motifs. Figure S2. Cell death analysis in tomato leaves. pART27:GFP, Csa4G642460 and Csa6G303740 were transiently expressed in Solanum lycopersicum. [file 12870_2020_2575_MOESM2_ESM.docx]

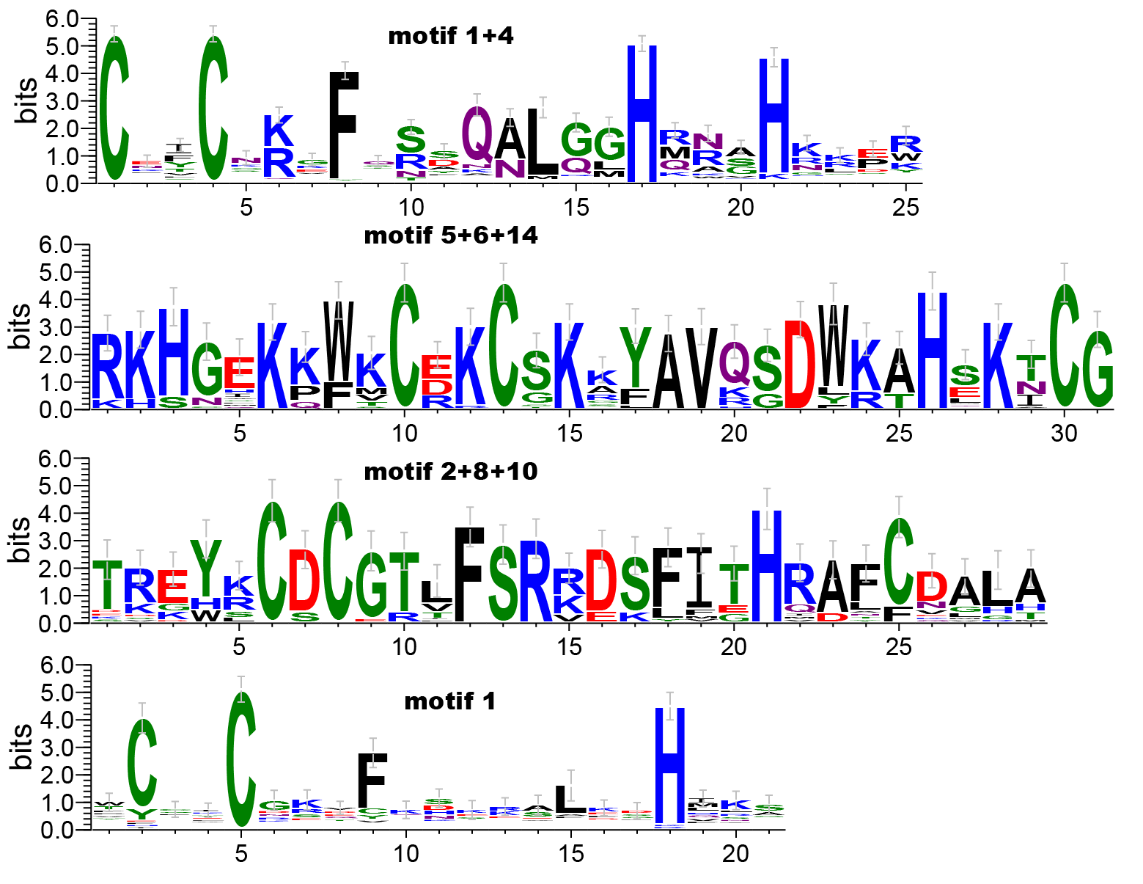


**Additional file 2: Figure S1.** The motif sequences and the conserved residuals in the motifs. The motif 1+4 and motif 5+6+12 represent the two typical C2H2 domains; the motif 2+8+10 represents the SprT-like zinc ribbon domain; the motif 1 represents the plant specific “QALGGH” domain.


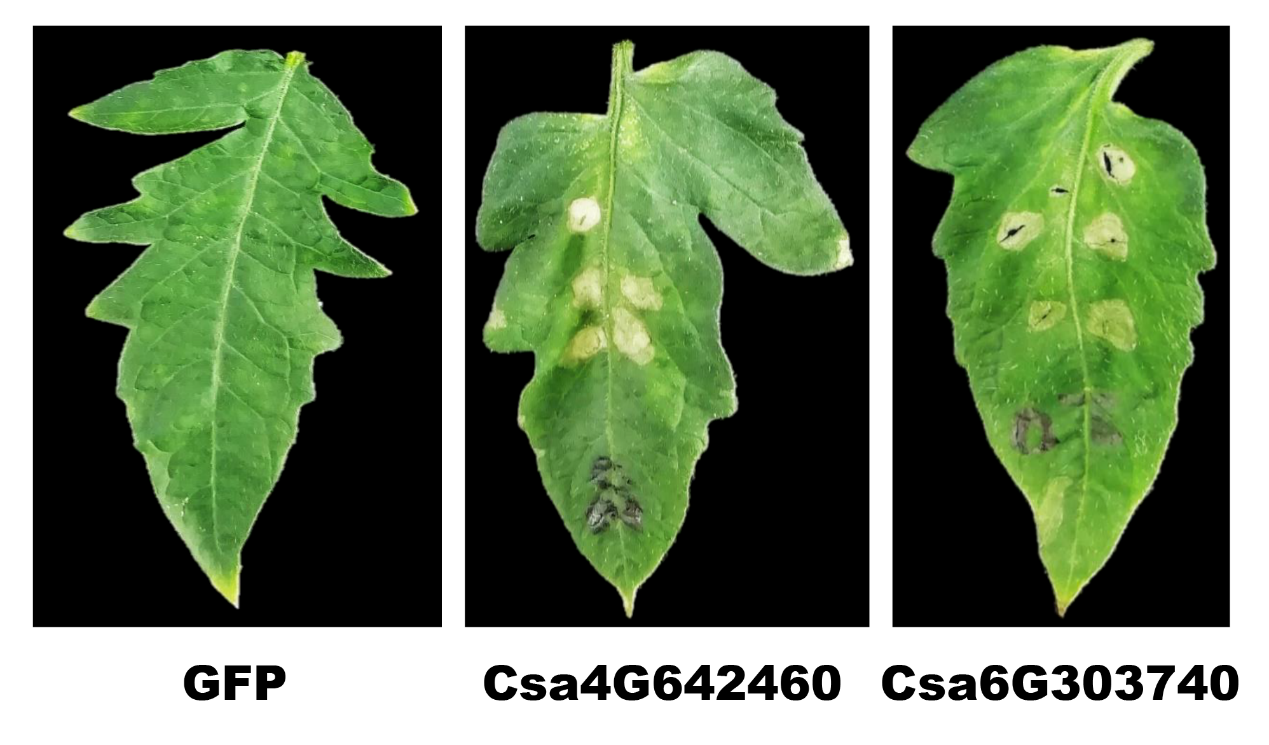


**Additional file 2: Figure S1.** Cell death analysis in tomato leaves. *pART27:GFP, Csa4G642460* and *Csa6G303740* were transiently expressed in *Solanum lycopersicum*.
